# Unravelling biocultural population structure in 4^th^/3^rd^ century BC Monterenzio Vecchio (Bologna, Italy) through a comparative analysis of strontium isotopes, non-metric dental evidence, and funerary practices

**DOI:** 10.1371/journal.pone.0193796

**Published:** 2018-03-28

**Authors:** Rita Sorrentino, Eugenio Bortolini, Federico Lugli, Giuseppe Mancuso, Laura Buti, Gregorio Oxilia, Antonino Vazzana, Carla Figus, Maria Cristina Serrangeli, Cristiana Margherita, Annachiara Penzo, Giorgio Gruppioni, Antonio Gottarelli, Klaus Peter Jochum, Maria Giovanna Belcastro, Anna Cipriani, Robin N. M. Feeney, Stefano Benazzi

**Affiliations:** 1 Department of Biological, Geological and Environmental Sciences–BiGeA, University of Bologna Via Selmi 3, Bologna, Italy; 2 Department of Cultural Heritage, University of Bologna, Via degli Ariani 1, Ravenna, Italy; 3 Complexity and Socio-Ecological Dynamics Research Group, Department of Humanities Universitat Pompeu FabraRamon Trias Fargas, Barcelona, Spain; 4 Department of Chemical and Geological Sciences, University of Modena and Reggio Emilia, Via Campi 103, Modena, Italy; 5 Department of Biology, University of Firenze, Via del Proconsolo, Florence, Italy; 6 UCD School of Medicine, Health Sciences Centre, University College Dublin Belfield, Dublin, Ireland; 7 Department of History and Cultures, University of Bologna, Piazza San Giovanni in Monte 2, Bologna, Italy; 8 Biogeochemistry Department and Climate Geochemistry Department, Max Planck Institute for Chemistry, Mainz, Germany; 9 Climate Geochemistry Department, Max Planck Institute for Chemistry, Mainz, Germany; 10 Lamont-Doherty Earth Observatory, Columbia University, Palisades, New York, United States of America; 11 Department of Human Evolution, Max Planck Institute for Evolutionary Anthropology, Deutscher Platz 6, Leipzig, Germany; University of Florence, ITALY

## Abstract

The 4^th^ century BC marks the main entrance of Celtic populations in northern Italy. Their arrival has been suggested based on the presence of Celtic customs in Etruscan mortuary contexts, yet up to now few bioarchaeological data have been examined to support or reject the arrival of these newcomers. Here we use strontium isotopes, non-metric dental traits and funerary patterns to unravel the biocultural structure of the necropolis of Monterenzio Vecchio (Bologna, Italy). Subsamples of our total sample of 38 individuals were analyzed based on different criteria characterizing the following analyses: 1) strontium isotope analysis to investigate migratory patterns and provenance; 2) non-metric dental traits to establish biological relationships between Monterenzio Vecchio, 13 Italian Iron age necropolises and three continental and non-continental Celtic necropolises; 3) grave goods which were statistically explored to detect possible patterns of cultural variability. The strontium isotopes results indicate the presence of local and non-local individuals, with some revealing patterns of mobility. The dental morphology reveals an affinity between Monterenzio Vecchio and Iron Age Italian samples. However, when the Monterenzio Vecchio sample is separated by isotopic results into locals and non-locals, the latter share affinity with the sample of non-continental Celts from Yorkshire (UK). Moreover, systematic analyses demonstrate that ethnic background does not retain measurable impact on the distribution of funerary elements. Our results confirm the migration of Celtic populations in Monterenzio as archaeologically hypothesized on the basis of the grave goods, followed by a high degree of cultural admixture between exogenous and endogenous traits. This contribution shows that combining different methods offers a more comprehensive perspective for the exploration of biocultural processes in past and present populations.

## Introduction

The ethnic and genetic background of northern Italy has been radically impacted by repeated invasions of central European Celtic populations from the 4^th^ century BC to the beginning of the 2^nd^ century BC. Such migratory processes began when members of different Celtic tribes crossed the Alps, while looking for unoccupied and disposable land to settle. According to ancient sources such as Polybius (Pol., II, 17), this migration was a traumatic experience for the local population, mostly involving Etruscans of the Po Valley, that often resulted in either the expulsion of indigenous populations or their subjugation [1–3].

Over the last few decades, great attention has been focused on the complex mechanisms of interaction between Celts and local populations in northern Italy during the two centuries preceding the Roman conquest. Both the archaeological record and epigraphic documentation from this period attests several types of interactions, such as replacement and/or extermination, as suggested by ancient historians (Dion. Hal., I, 18, 5; Pol., II, 17; Serv., Ad Aen., X, 168). The graveyards linked to Celtic presence in this area (in particular in the area surrounding the town of Bologna) are pivotal for understanding the patterns of interaction between local Etruscans and the incoming Celts. Archaeological data are even more explicit than epigraphic sources in suggesting that processes of interaction between local Etruscan and Celtic population ranged from conflict–well exemplified by sites such as the necropolis "Zone A" at Casalecchio di Reno (Bologna) and Marzabotto (Bologna)–to cases of less problematic and more rapid integration documented at sites such as Felsina (Etruscan Bologna) and Monte Bibele on the Apennines to the south of Bologna [1,2,4–7].

Archaeological data reveal that with the arrival of the Celts, “Zone A” at Casalecchio di Reno was abandoned by Etruscans in the early decades of the 4^th^ century, and was then occupied by the Celts three-quarters of a century later [8–11]. The Celts located their necropolis upon the Etruscan ruins, using typical Celtic rituals and funeral customs (i.e., inhumation instead of incineration, and the widespread presence of weapons in the graves) [12]. The hiatus caused by the Celtic invasion is even more evident at the urban site of Marzabotto, where the entire settlement area was used for funerary purpose (i.e., wells were used as burials, while rooms of houses were used as enclosures) [13]. Felsina probably experienced less traumatic events, as the city maintained a prominent position within the territory even during the Celtic period. However, the earliest phase of the invasion (end of the 5^th^ century BC) was characterized by a general impoverishment and considerable change in the urban setting, including wells used as burials (as in the case reported for Marzabotto). Following this initial period, archaeological evidence uncovered at Felsina show sign of gradual integration between the two ethnic groups, primarily suggested by the coexistence of both traditional Celtic and Etruscan funerary practices. The same patterns of coexistence were observed in the necropolises of Monte Bibele and Monterenzio Vecchio (Bologna) [5,13].

The necropolis of Monte Bibele is well known as the largest “Celtic cemetery” in Italy, comprising at least 171 graves which were thoroughly studied from both archaeological and anthropological perspectives [12,14–18]. According to these investigations, the necropolis can be dated to an interval comprising the end of the 5th and the middle 3rd century BC. The funerary area is characterized by the simultaneous presence of objects ascribable to both Etruscan and Celtic traditions, in addition to objects possibly deriving from an actual process of admixture between these two traditions [12,16]. The presence of local (likely Etruscan) and non-local (likely Celtic) groups at the site was independently and more recently confirmed by isotope analysis [19].

Unlike Monte Bibele, the nearby necropolis of Monterenzio Vecchio was never investigated with the same intensity. In the graves of Monterenzio Vecchio, cultural syncretism between Celts and Etruscan indigenous peoples was manifested in the funerary behaviour by the intertwining of northern Alpine customs (funerary ideology dominated by weapons) and the local Mediterranean tradition (ideology of the banquet). This process of exchange and cultural admixture resulted in the development of an Etruscan-Celtic culture at early 4^th^ century BC [20–22].

**Fig 1:**
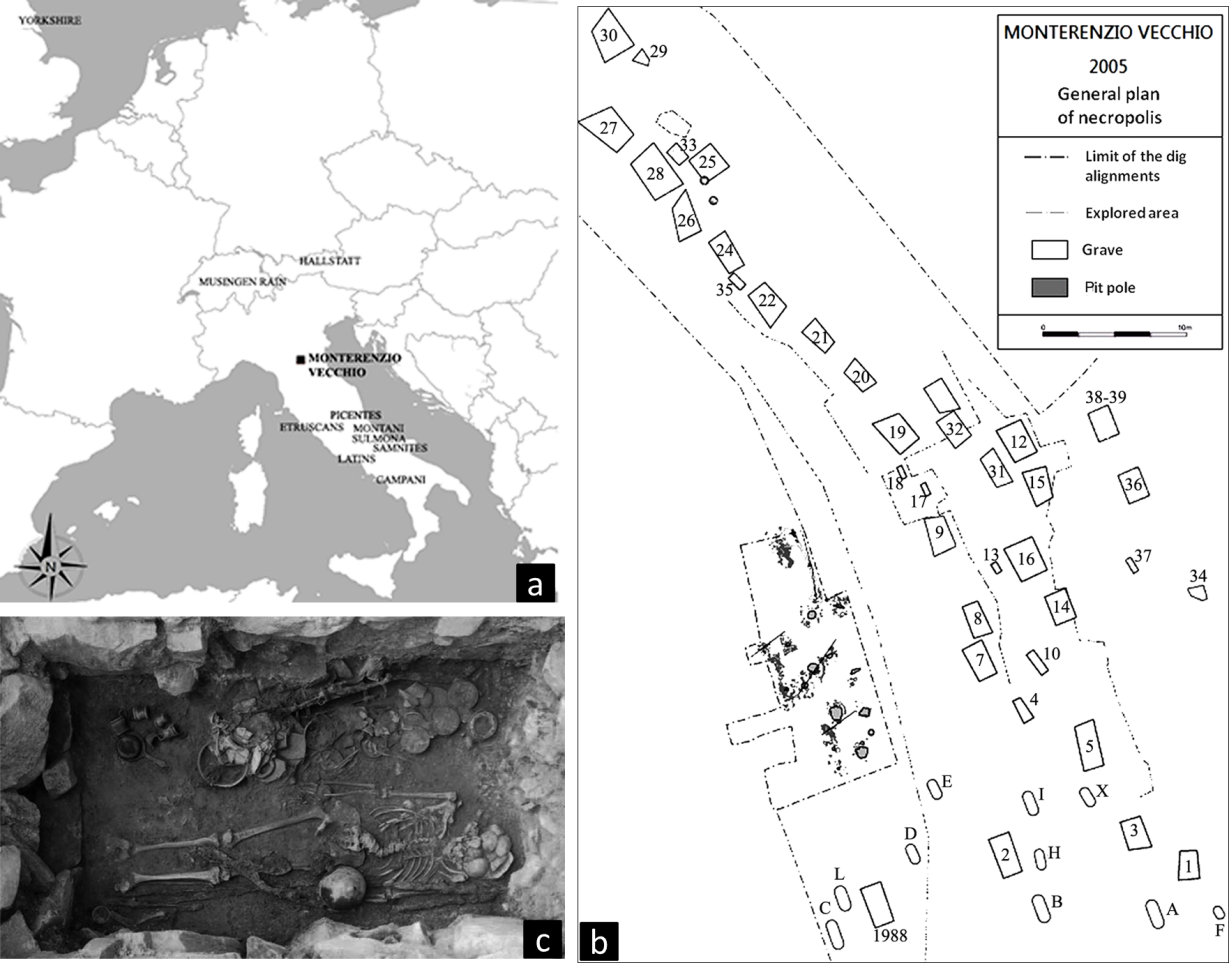
a) Location of Monterenzio Vecchio in relation to the Italian and Celtic comparative sample. b) Plan of the Etruscan-Celtic necropolis of Monterenzio Vecchio (Cartographic Archive—Museum "Luigi Fantini" of Monterenzio). c) Grave 36: adult male burial with rich grave goods (Photo archive of the Museum "L. Fantini" of Monterenzio).

Paradoxically, up to now there have been no attempts to use biocultural data to evaluate whether the Celtic populations had arrived and occupied Etruscan settlements. More specifically, even though cultural data strongly suggest the arrival of newcomers, there have been no biocultural attempts to confirm the presence of a potential Celtic component in local Etruscan communities. Due to the importance of migrations and interactions of different ethnic groups that shape the culture and biology of later populations [19,23–29], in this paper we use strontium isotope ratio, non-metric dental features and funerary data to investigate the biocultural structure of the necropolis of Monterenzio Vecchio (Fig 1) during this critical timeframe of cultural migrations.

Our goals are three-fold: 1) to assess potential local and non-local individuals and investigate mobility patterns among individuals of Monterenzio Vecchio by recording variations in strontium isotope ratios of human and animal bones and teeth; 2) to perform a regional analysis based on non-metric dental traits to verify the biological distance among Monterenzio Vecchio, Celts (continental and non-continental samples described by Anctil, 2016 [30]) and other Iron Age Italian populations [31], ultimately to assess whether a potential contribution of Celtic newcomers may have affected the biological background at Monterenzio Vecchio; 3) to detect the possible cultural population structure by means of statistical modeling of funerary indicators.

Sr isotopes are a strong tool to detect past human migratory patterns and provenance [32]. The local soils and water retain a particular Sr isotope signature (^87^Sr/^86^Sr), which derives from the local bedrock, and, in turn, is related to petrogenetic processes, age and initial rubidium content of the rock itself, given that ^87^Rb decays into ^87^Sr through time. Leached strontium ions enter the ecosystem and build up the reservoir of bioavailable Sr, and following the trophic chain are fixed in the hydroxyapatite of bones and teeth replacing some of the calcium. During this path from the bedrock to the skeletal tissue, the ^87^Sr/^86^Sr ratio of the bioavailable Sr does not change, and therefore the Sr isotope composition of the skeletal tissue replicates that of the living location, allowing for the detection the movements of an individual during their life between geologically different regions. In particular, given that tooth enamel does not remodel after the mineralization phase and is quite resistant to diagenetic alteration, its Sr isotopic composition reflects the ^87^Sr/^86^Sr ratio of the location where the individual spent their childhood. Thus, through a comparison of the ^87^Sr/^86^Sr ratio between tooth enamel and bioavailable strontium from the archeological site area, it is possible to discriminate between local and non-local individuals [32]. However, it is important to note the limitations of this technique. When individuals eat foods that are imported from an isotopically different region for instance, the signature mimics a non-local origin based on isotopes. In a similar light, it is not possible to distinguish between two regions with isotopically similar geology.

We used non-metric dental traits to establish phenotypic variability and biological relationships between populations [25,28,30,31,33–42]. These traits bear a strong genetic background, as confirmed by ancient DNA [43–45], are numerous, and are generally not correlated with sex or age [28,33,36,41,46–50]. Assuming that individuals or populations with a similar pattern of non-metric dental variables are more closely biologically related than those with different patterns, the inter-biodistance analysis (i.e., among Monterenzio Vecchio, and the comparative sample of Celts and Iron Age Italian necropolis) is a useful method to identify the biological background of Monterenzio Vecchio.

Over the past thirty years, approaches to archaeology and anthropology have been inspired by cultural evolutionary theory (or Dual Inheritance Theory) [51,52]. Such theory has been investigating the mechanisms responsible for culture change over time and space and the processes responsible for the transmission of cultural information at different scales. In particular, with rapid development and reduced costs of genetic and palaeogenetic analyses, greater attention has been devoted to co-evolutionary patterns between genetic/morphological/ancestral backgrounds and the distribution of a variety of cultural variants (e.g., [53–59]). In particular, these studies aim to understand whether the observed distribution of cultural elements across populations can be mostly ascribed to the movement of people (demic diffusion), or rather is the result of more composite scenarios dominated by the transmission of cultural information without necessarily entailing population-level moments (cultural diffusion). However, to this point, there has been only limited study of the differential rates at which genetic and cultural information may be exchanged in contexts of documented population movement. In this framework, Monterenzio Vecchio offers a unique opportunity to thoroughly study mechanisms of cultural transmission and cultural admixture in relation to an independently documented event of population movement and replacement (demic diffusion). Overall, this approach aims to select the most likely process of interaction between local and non-local populations among models proposed in the literature, ranging from total replacement of local customs by non-local ones to the generation of a novel and completely admixed form of material culture.

The application of these three approaches (non-metric dental traits, strontium isotopes and cultural modeling) will maximize the likelihood of confirming/rejecting the hypothesis about migration of Celtic groups in Monterenzio Vecchio and will improve our understanding of the cultural processes that occurred during the interaction between the newcomers (if confirmed) and the indigenous populations.

### Archaeological context

The Etruscan-Celtic necropolis of Monterenzio Vecchio (578 MASL) is located in the Tuscan-Emilian Apennines, 30 kilometers southeast of Bologna (Italy), on the east side of the Idice Valley. Its elevated position demonstrates the importance of the valley as a strategic center for military, economic and social purposes, and Monterenzio Vecchio is an outpost that allowed its inhabitants to control the transit between Romagna and Tuscany, such as in the case of the Roman advancement towards northern Italy. Further evidence of the site importance is the presence of the *Flamina Minor* consular route established in 187 BC, which connected Bologna to Arezzo, created to contrast these Celtic military settlements. The necropolis of Monterenzio Vecchio (Fig 1B) was discovered fortuitously in 1882. However, it was only in 1988 that an emergency excavation led to the discovery of at least 12 disturbed graves (there is uncertainty about the exact number of graves discovered during this year), some of which were referred to as pits, while others were represented only by scattered human remains. The systematic exploration of the area was undertaken between 2000 and 2005 by the Department of Archaeology of the University of Bologna, in collaboration with the École Française de Rome and the CNRS UMR 8564 (ENS), Paris. Five years of excavation revealed the presence of 38 graves, which when added to the 1988 findings totaled 50 graves. The grave goods (some examples are listed below) are dated to the La Tène B2 period, or the Second Iron Age, i.e., to the 4^th^–3^rd^ century BC based on typological and stratigraphic comparisons [20,21,59].

The funerary area is spread along the hillside, and the graves are aligned across several artificial terraces. Some graves were empty with no skeletal remains or grave goods, while others were only partially disturbed. Inhumation is the most frequent funerary practice, while cremation is documented in only four cases. Adults were buried in large, carefully built rectangular pits (withaverage dimensions between 2–3 meters in length x 1–2.30 meters wide), while the children’s graves were less well-defined. The funerary structures consisted of external stone walls and an internal wooden structure, possibly used as a burial chamber. In some cases, the presence of grave goods underneath the body suggests that a wooden stretcher was used as a support for the dead. The deceased was placed in a supine position along the eastern side of the grave with the head invariably facing north, while the less frequent cremated remains were probably placed inside containers of organic, perishable materials [20,21,60].

The different objects that served as funerary goods have been hypothesized to be the result of the intensive interaction between local Etruscans and members of the incoming Celtic tribes [20,21,59]. Adults were always accompanied by a rich set of grave goods (Fig 1C), such as banquet vessels (usually ceramics, rarely in bronze), and banquet-related items (spits and knives), food offerings, transalpine weapons (iron sword with scabbard, spear and shield), Italic weapons (helmets and long spears), grooming tools for males (strigils, shears, and razors) and females (bronze mirrors), and female-associated artifacts including sewing implements and jewelry sets (necklaces made of amber or glass beads, silver brooches, clasp/*fibulae*). Children do not seem to exhibit grave goods which are specific for their age, although these items are usually smaller than those assigned to adults [20,21]. Therefore, specific attributes are considered as diagnostic of each ethnic group. Iron sword with scabbard, spear and shield are believed to be usually associated with males of Celtic origins, while long spears (*pilum*) might reflect the Etruscan culture. One of the most debated element is the helmet which can be considered the emblema of the Celtic/Etruscan syncretism in Monterenzio Vecchio. The design of the helmet capes is Etruscan/Italic (Montefortino type), whereas the botton appliances on the helmet seem to be of Celtic origin. Bronze mirrors have been linked in the literature to Etruscan females, whereas clasp/*fibulae* may belong to either the Certosa (Etruscan) or the La Tène (Celtic) faces [20,21,59]. The consistent and ubiquitous presence of both local and non local elements in the vast majority of tombs dated to the 4^th^ and 3^rd^ centuries BC suggests that Monterenzio Vecchio could be a good example of the arrival of new cultural markers with the physical arrival of newcomers. Etruscan and Celtic demes were part of a long process of cultural admixture at Monterenzio Vecchio. As Etruscan and Celtic artifacts might be present in graves dating to the 4^th^ and 3^rd^ century BC, a cultural syncretism (at least for the funerary behavior) has been suggested [13,21,61].

## Materials and methods

### Sample

The necropolis of Monterenzio Vecchio consists of 50 graves, with only 38 graves yielding well preserved archaeological artifacts and human remains. No permits were required for the described study since access to materials was granted by AG, Director of Museo Civico Archeologico L.Fantini, and member of the Department of History and Cultures, University of Bologna, which is the only beneficiary of concessions for excavation, study of materials, publication of results, and management of the Museum. All specimens investigated in the present study are stored at Museo Civico Archeologico L.Fantini of Monterenzio (Bologna, Italy). The materials can be accessed upon request presented to the Museum Director (museomonterenzio@unibo.it).

Our sample comprises a total of 38 individuals, with sub-samples selected depending on the criteria characterizing each analysis in this study (see Table 1).

**Table 1:** Individuals analyzed from the Monterenzio Vecchio necropolis.

Individual	Sex^a^	Age^b^	Ritual	Strontium isotopes	Non-metric dental trait	Funerary data
MV 1	M	MA	inhumation	x	x	x
MV 2	F	MA	inhumation	x	x	x
MV 3	M	YA	inhumation	x	x	x
MV 4	F	OA	inhumation	-	-	x
MV 5	M	OA	inhumation	x	x	x
MV 6	-	-	cremation	-	-	x
MV 7	PF	OA	inhumation	x	x	x
MV 8	M	MA	inhumation	x	x	x
MV 9	F	OA	inhumation	-	-	x
MV 10	-	C	inhumation	x	-	x
MV 12	M	YA	inhumation	x	x	x
MV13	-	I	inhumation	-	-	x
MV 14	F	OA	inhumation	-	-	x
MV 15	M	MA	inhumation	x	x	x
MV 16	-	-	cremation	-	-	x
MV 17	-	I	inhumation	-	-	x
MV 18	-	C	inhumation	-	-	x
MV 19	M	YA	inhumation	x	x	x
MV 20	F	OA	inhumation	x	x	x
MV 21	F	MA	inhumation	x	x	x
MV 22	M	A	inhumation	x	x	x
MV 23	-	I	inhumation	-	-	x
MV 24	-	C	inhumation	x	-	x
MV 25	-	C	inhumation	x	-	x
MV 26	M	YA	inhumation	x	x	x
MV 27	M	YA	inhumation	x	x	x
MV 28	-	-	cremation	-	-	x
MV 29	-	C	inhumation	-	-	x
MV 30	M	A	inhumation	x	x	x
MV 31	F	OA	inhumation	x	-	x
MV 32	-	-	cremation	-	-	x
MV 33	-	C	inhumation	-	-	x
MV 35	-	C	inhumation	x	-	x
MV 36	M	MA	inhumation	-	x	x
MV 37	-	C	inhumation	-	-	x
MV 39	M	YA	inhumation	x	x	x
MV TA	F	YA	inhumation	x	x	-
MV TB	M	YA	inhumation	x	x	-

^a^Sex: M: male; F: female; PF: probable female.

^b^Age categories: I: infant (0–3 years); C: child (3–12 years); A: adolescent (12–20 years); YA: young adult (20–35 years); MA: middle adult (35–50 years); OA: old adult (>50 years).

Skeletal samples were examined in the Laboratory of Physical Anthropology, Department of Cultural Heritage (Ravenna), University of Bologna. The sex of the individuals was assessed using established pelvic [62] and cranial morphological methods [63]. Age at death was estimated by a combination of methods [63–69], and all individuals were assigned to age cohorts according to the standards described by Buikstra and Ubelaker [70].

### Strontium isotope chemical preparation and analysis

A total sample of 44 teeth, collected from 23 individuals, was sampled for strontium isotope analysis (Table 2). Two teeth were sampled per individual, with the exception of two cases where only one tooth was available (M_1_ for individual MV1 and M_3_ for individual MV5). For each individual, except MV1 and MV5, the available tooth that mineralized first was used to evaluate local versus non-local individuals, while the available tooth that mineralized last in the sequence was used to evaluate the potential mobility pattern of the individual during life. In addition, a sample of five human skeletal elements were collected to evaluate the local strontium isotopic range of Monterenzio Vecchio.

**Table 2:** 87Sr/86Sr ratio of the individuals from Monterenzio Vecchio.

Individual	Material	Tooth sampled	^87^Sr/^86^Sr	2SE*
MV 1	enamel	LM_1_	0.708856	0.000005
MV 2	enamel	RM^2^	0.708974	0.000006
	enamel	RM_3_	0.709032	0.000006
MV 3	enamel	LM_1_	0.708977	0.000007
	enamel	RM^3^	0.708779	0.000007
MV 5	enamel	LM_3_	0.709108	0.000006
MV 7	enamel	LM_1_	0.708896	0.000007
	enamel	LM^2^	0.709337	0.000006
MV 8	bone	-	0.708733	0.000006
	enamel	LM^1^	0.708914	0.000007
	enamel	RM_3_	0.708838	0.000005
MV 10	enamel	Ldm_2_	0.708793	0.000006
	enamel	RM_1_	0.708866	0.000006
MV 12	enamel	RM^1^	0.709257	0.000007
	enamel	RM_2_	0.708980	0.000005
MV 15	enamel	LM_1_	0.709266	0.000005
	enamel	RM_3_	0.709319	0.000007
MV 19	enamel	LM_1_	0.709070	0.000006
	enamel	LM_3_	0.709192	0.000006
MV 20	enamel	LM_1_	0.709478	0.000008
	enamel	LM_2_	0.709482	0.000005
MV 21	enamel	LP^4^	0.708501	0.000008
	enamel	RM_1_	0.708533	0.000007
MV 22	bone	-	0.708685	0.000007
	enamel	LP_4_	0.709043	0.000006
	enamel	RM_1_	0.709032	0.000006
MV 24	enamel	Rdm_2_	0.708757	0.000007
	enamel	RM_1_	0.708814	0.000006
MV 25	enamel	Rdm_2_	0.708755	0.000005
	enamel	RM_1_	0.708810	0.000005
MV 26	bone	-	0.708701	0.000007
	enamel	LM_1_	0.709143	0.000006
	enamel	LM_2_	0.708997	0.000004
MV 27	bone	-	0.708691	0.000007
	enamel	RM^1^	0.709354	0.000008
	enamel	RM^3^	0.708812	0.000005
MV 30	enamel	LM^1^	0.708944	0.000007
	enamel	RM^3^	0.708743	0.000005
MV 31	bone	-	0.708826	0.000007
	enamel	LM_1_	0.709363	0.000007
	enamel	LM_3_	0.709289	0.000004
MV 35	enamel	Ldm^2^	0.708806	0.000006
	enamel	LM_1_	0.708740	0.000005
MV 39	enamel	LM^1^	0.708763	0.000005
	enamel	RM_3_	0.708749	0.000005
MV A	enamel	LM^3^	0.708850	0.000006
	enamel	RM^1^	0.709101	0.000004
MV B	enamel	LM^1^	0.708879	0.000006
	enamel	LM_2_	0.708947	0.000005

*2SE: 2 Standard Error

Sample preparation and column chemistry were performed at the Max Planck Institute for Chemistry (Mainz, Germany), following the protocol described in Lugli et al., 2017 [71].

Small fragments of tooth enamel were separated from the dentine using a dentist drill. Enamel and bone samples were then ultrasonically washed with MilliQ® and leached with 0.2N HNO3. Samples were digested in 1ml of suprapur 14N HNO3 and evaporated to dryness (at100° C). The residues were digested in a droplet of 14N HNO3 plus a droplet of suprapur H2O2 (30%), and left to dry on a hotplate at 100°C. This process was repeated until all the organic matter was oxidized. Samples were finally re-dissolved in 3ml of 3N HNO3 and after centrifuging were loaded into 300 μl volume columns filled with Eichrom Sr spec–resin and washed with 3N HNO3. Sr was then eluted with several reservoirs of MilliQ® water [71].

The ^87^Sr/^86^Sr isotopic composition of the samples, diluted to 200 ppb, was determined using a double focusing MC–ICP–MS with a forward Nier–Johnson geometry (Thermo Fisher Scientific, Neptune™) housed at the Centro Interdipartimentale Grandi Strumenti (CIGS) of the University of Modena and Reggio Emilia [72,73]. A bracketing sequence [blank/standard/blank/sample/blank] has been used to monitor any eventual drift of the instrument. Sr blanks were lower than 20 pg. The ^87^Sr/^86^Sr ratios have been corrected for instrumental bias to an NBS–987 value of 0.710260 [74]. Repeated analyses of the NBS–987 yielded a ^87^Sr/^86^Sr ratio of 0.710302 ± 0.000020 (2σ; n = 39).

### Non-metric dental trait recording and analysis

From an original sample of 23 adult individuals (total number of individuals excluding cremations and sub-adults) from Monterenzio Vecchio, with well-preserved permanent teeth and free from dental pathological conditions (e.g., caries, calculus and antemortem tooth loss), four individuals (MV 4, MV9, MV 14, MV 31) were removed from analysis on account of a reduced number of teeth and/or an excessive amount of missing data. Missing data may be due to ante- or post mortem tooth loss, dental damage by pathological conditions (e.g., caries, calculus), and heavy dental wear that can obscure traits, ultimately leading to a sample size reduction [75,76]. A list of individuals included in the analysis is reported in Table 1.

Dental and oral osseous traits from 19 individuals of Monterenzio Vecchio were recorded following the Arizona State University Dental Anthropology System (ASUDAS) [50] in order to assess the inter-group affinity of the Monterenzio Vecchio sample with published data from three continental and non-continental Celtic necropolises [30] and from 13 Italian Iron Age necropolises [31] (Fig 1A, Table 3). In particular the Celtic sample consists in proto-Celts (650–475 BC—Hallstatt D, HalD) from Hallstatt (Austria), continental Munsingen-Rain (420–240 BC—La Tène A, B, C, MunRain) from Munsingen (Switzerland) and non-continental Celts from Yorkshire (400–100 BC—La Tène A, B, and the first half of C, BRIT) in England. The 13 Iron Age necropolises from central-southern Italy (up to now, non-metric dental traits for Iron Age necropolis located in northern Italy are not available) were organized in three main periods: A (early 9^th^–8^th^ century BC), B (middle 7^th^–5^th^ century BC), and C (late 4^th^–2^nd^ century BC). The sample includes Latins (LAA, LAB, LAC), Etruscans (ETB, ETC), Picentes (PCB, PCC), Campani (CAA, CAB, CAC), Samnites (SAM), Montani (MON) and Sulmona (SUL).

**Table 3:** Samples used in the analysis of regional biological distance.

Group	Sample	Abbreviation	Chronological periods^4^	Number of individuals
*Italics*^*1*^	Ancient Etruscans	ETB	B	95
	Recent Etruscans	ETC	C	102
	Archaic Latini	LAA	A	33
	Ancient Latini	LAB	B	41
	Recent Latini	LAC	C	94
	Ancient Piceni	PCB	B	136
	Recent Piceni	PCC	C	211
	Montani	MON	A/B	40
	Sulmona	SUL	C	52
	Samnites	SAM	B	163
	Archaic Campani	CAA	A	65
	Ancient Campani	CAB	B	28
	Recent Campani	CAC	C	54
*Celts*^*2*^	Hallstatt	HalD	Hallstatt D	30
	Munsingen-Rain	MunRain	La Tène A, B, C	33
	Yorkshire	BRIT	La Tène A, B, first half of C	31
*Italic/Celts*^*3*^	Monterenzio Vecchio	MV	La Tène B2	19
	*Total*			*1245*

^1^Italic data are taken from Coppa et al.(1998).

^2^Celtic sample presented in Anctil (2016).

^3^Italic/Celts listed in this study.

^4^A: early 9th to 8th century BC; B: middle 7th to 5th century BC; C: late 4th to 2nd century BC; Hallstatt D: middle 7th to early 5th century BC; La Tène: 4th to 1st century BC.

For the comparison, Monterenzio Vecchio was considered as a whole (all individuals of Monterenzio Vecchio: MV), and was subsequently separated according to the isotopic results in local (MVL) and non-local (MVNL) individuals.

Although a total of 42 discrete dental and oral osseous traits were initially collected for each individual of Monterenzio Vecchio, only 14 traits were evaluated in the present work (Table 4) due to limited overlap in observed traits across samples and in the breakpoints adopted in different datasets [30,31]. Dental traits were observed macroscopically on left-side dentitions according to rank-scales of expression [50]. When the left tooth was missing the antimere was used. Trait scores were converted to either “present” or “absent” using dichotomous breakpoints of the comparative sample [30,31] and then converted into frequencies. Trait frequencies were used to carry out the Mean Measure of Divergence (MMD), coupled with the Freeman and Turkey correction for very low or very high trait frequencies and small sample sizes [77–81]. MMD calculates between sample biological divergence, thus yielding a dissimilarity measure between each sample pair, where low MMD values indicate similarity and higher ones indicate distance [36,37,77,80,82]. Although a more precise MMD estimation requires the use of as many discrete traits as possible, the traits should all be independent from one another [80]. Thus, Pearson chi-square analysis on trait proportion was used to find and exclude highly correlated traits (r > 0.5). Significant differences among samples is found for MMD > 2SD, rejecting the null hypothesis (P1 = P2, P = sample population) at the P<0.025 significance level [77,80,83]. Finally, multidimensional scaling (MDS) and hierarchical cluster analysis (Ward’s method) on a MMD pairwise distance matrix were used to further investigate the relationship between samples. The data were processed through software routines written in R version 3.3.0 [84].

**Table 4:** Percentages (%) of discrete dental traits and number of individuals (n) of in the samples.

Traits		MV	MVL	MVNL	BRIT	MunRain	HalD	LAA	LAB	LAC	ETB	ETC	PCB	PCC	CAA	CAB	CAC	SUL	SAM	MON
Double shovel UI1*	*%*	11.1	0.0	20.0	0.0	0.0	0.0	12.5	0.0	0.0	16.7	9.1	15.6	0.0	14.3	0.0	4.8	8.3	3.3	0.0
(+ = ASU 2–6)	*n*	18	7	10	31	33	30	8	5	19	6	11	32	41	14	7	21	12	30	9
Interruption groove UI2	*%*	44.4	85.7	20.0	38.7	30.3	40.0	66.7	75.0	63.6	54.5	70.8	77.5	65.8	63.2	90.9	30.4	58.3	65.5	64.7
(+ = ASU +)		18	7	10	31	33	30	9	8	22	11	24	40	73	19	11	23	12	58	17
Tuberculum dentale UI2*	*%*	47.1	57.1	44.4	58.1	60.6	30.0	66.7	42.9	65.4	50.0	43.5	62.2	57.5	65.0	53.8	24.0	60.0	50.0	58.8
(+ = ASU 2–6)	*n*	17	7	9	31	33	30	9	7	26	10	23	45	80	20	13	25	10	50	17
Distal accessory ridge UC*	*%*	25.0	33.3	20.0	29.0	57.6	33.3	33.3	62.5	70.4	81.8	58.3	73.0	66.1	92.9	60.0	66.7	63.6	74.3	57.1
(+ = ASU 2–5)	*n*	8	3	5	31	33	30	9	8	27	11	12	37	59	14	5	15	11	35	14
Carabelli's trait UM1*	*%*	43.8	71.4	25.0	25.8	24.2	10.0	71.4	62.5	78.6	58.2	50.0	58.2	62.8	70.8	53.8	48.3	37.5	71.7	63.2
(+ = ASU 2–7)	*n*	16	7	8	31	33	30	14	16	42	12	20	55	94	24	13	29	16	60	19
Parastyle UM3*	*%*	15.4	0.0	40.0	25.8	27.3	20.0	40.0	6.7	10.0	0.0	10.0	23.8	20.3	30.0	9.1	13.8	0.0	8.2	0.0
(+ = ASU 1–5)	*n*	13	7	5	31	33	30	10	15	40	18	20	42	69	20	11	29	12	49	20
Premolar root number UP1*	*%*	42.9	60.0	37.5	32.3	27.3	43.3	50.0	60.0	0.0	73.3	57.1	63.6	69.8	47.1	81.8	47.4	63.6	78.3	52.6
(+ = ASU 2+)	*n*	14	5	8	31	33	30	6	10	2	15	28	55	86	17	11	19	22	46	19
Molar root number UM2*	*%*	94.1	100.0	88.9	35.5	30.3	63.3	70.0	62.5	75.0	73.7	69.6	72.9	69.1	84.2	84.2	69.2	44.4	73.5	61.1
(+ = ASU 3+)	*n*	17	7	9	31	33	30	10	8	4	19	23	48	81	19	19	13	18	49	18
Lingual cusp LP2*	*%*	72.2	75.0	77.8	61.3	27.3	23.3	50.0	41.7	54.3	24.0	39.4	43.8	46.2	65.0	58.3	37.8	58.3	62.7	36.8
(+ = ASU 2–9)	*n*	18	8	9	31	33	30	12	12	35	25	33	48	65	20	12	37	12	59	19
Groove pattern LM2*	*%*	7.1	12.5	0.0	6.5	0.0	36.7	26.7	44.4	27.8	33.3	19.6	28.1	23.6	39.4	23.5	15.2	13.6	12.7	16.0
(+ = ASU Y)	*n*	14	8	5	31	33	30	15	18	36	36	51	64	110	33	17	33	22	63	25
Cusp number LM1*	*%*	0.0	0.0	0.0	6.5	0.0	16.7	0.0	6.2	8.1	3.2	2.2	2.3	3.2	12.1	0.0	0.0	9.1	1.4	0.0
(+ = ASU 6+)	*n*	18	8	9	31	33	30	13	14	34	31	45	44	93	33	16	25	22	69	23
Deflecting wrinkle LM1	*%*	0.0	0.0	0.0	25.8	54.5	20.0	12.5	10.0	10.0	0.0	12.5	11.4	15.6	21.1	0.0	25.0	11.1	5.0	12.5
(+ = ASU 2–3)	*n*	2	0	2	31	33	30	8	10	20	15	8	35	45	19	5	8	9	20	8
Trigonid crest LM1	*%*	0.0	0.0	0.0	29.0	42.4	16.7	0.0	7.7	6.7	5.9	7.7	4.3	11.1	0.0	0.0	16.7	8.3	2.9	0.0
(+ = ASU +)	*n*	4	3	1	31	33	30	9	13	30	17	13	46	63	26	8	12	12	35	19
Protostylid LM1	*%*	6.7	0.0	14.3	19.4	27.3	23.3	81.8	85.7	74.2	62.5	48.5	74.5	75.3	65.4	63.6	10.5	58.8	65.9	93.8
(+ = ASU 1–6)	*n*	15	7	7	31	33	30	11	14	31	24	33	51	81	26	11	38	17	44	16

*Traits used in MMD analysis.

### Systematic description and analysis of archaeological data

To analyze the distribution and variability among the grave goods at Monterenzio Vecchio, evidence on funerary practices was first collected from unpublished materials, so that all 36 individuals could be directly compared across the same set of items (S1 Table). Then each individual was described by a string of mutually exclusive, categorical values expressing presence (1) or absence (0) for each cultural marker. Inter-individual distance was computed as a Jaccard distance [85,86] and the impact of variables such as sex dimorphism, age, and provenance (inferred from isotope results) was first qualitatively assessed through a complete-linkage hierarchical clustering [87]. Quantitative assessment of the impact of grouping according to the same variables (sex, age and origin) was first conducted via Correspondence Analysis [88], and more formally investigated through Analysis of Molecular Variance (AMOVA) [89,90] to infer the amount of inter-group variation as opposed to the amount of intra-group variation (the resulting summary statistic, Φ_ST_, is the ratio between inter-group variation and total variation measured in the metapopulation).

AMOVA can be directly computed on symmetric distance matrices and has been already employed to measure population structure based on cultural markers [54,91–93], yielding significant Φ_ST_ values comprised between 0.02 and 0.13.

The Random Forest [94–97] algorithm was employed in the present case to identify cultural variables that are more likely to correctly classify individuals based on their sex or on their provenance. This supervised learning technique uses recursive binary splitting to grow classification trees. Variable choice at each node depends on a variety of measures of classification error, such as Classification Error Rate (the maximum portion of observations that do not belong to the most common class) or Gini index (the sum across all classes of the products between class evenness and class error rate computed at each node). To add accuracy to the process, multiple sampling with replacement is carried out at each node (i.e., bootstrap aggregation), where individual predictive models are computed for each subset and then averaged by choosing the most commonly occurring model among all predictions. In addition, at each node Random Forest randomly selects a subset of predictors (whose number usually approximates the square root of the total number of predictors), further reducing the impact of correlation between variables and the impact of strong predictors.

Once variables that were most likely to be linked with sex were identified and taken out, the strong collinearity remaining in the dataset was addressed using the source code provided by Marcus W. Beck on GitHub (https://gist.github.com/fawda123/4717702), which performs a stepwise variable selection based on calculation of variance inflation factors (VIF), and isolates the variables that exhibit VIF value below a given threshold (5 for the present study). Variance inflation of a given variable is computed as the reciprocal of the inverse of the coefficient of determination (R^2^), expressing the correlation between that variable and all other variables in a matrix.

All datasets were processed and analyzed through software routines written in R version 3.3.0 [84].

## Results

### Strontium isotope

**Fig 2:**
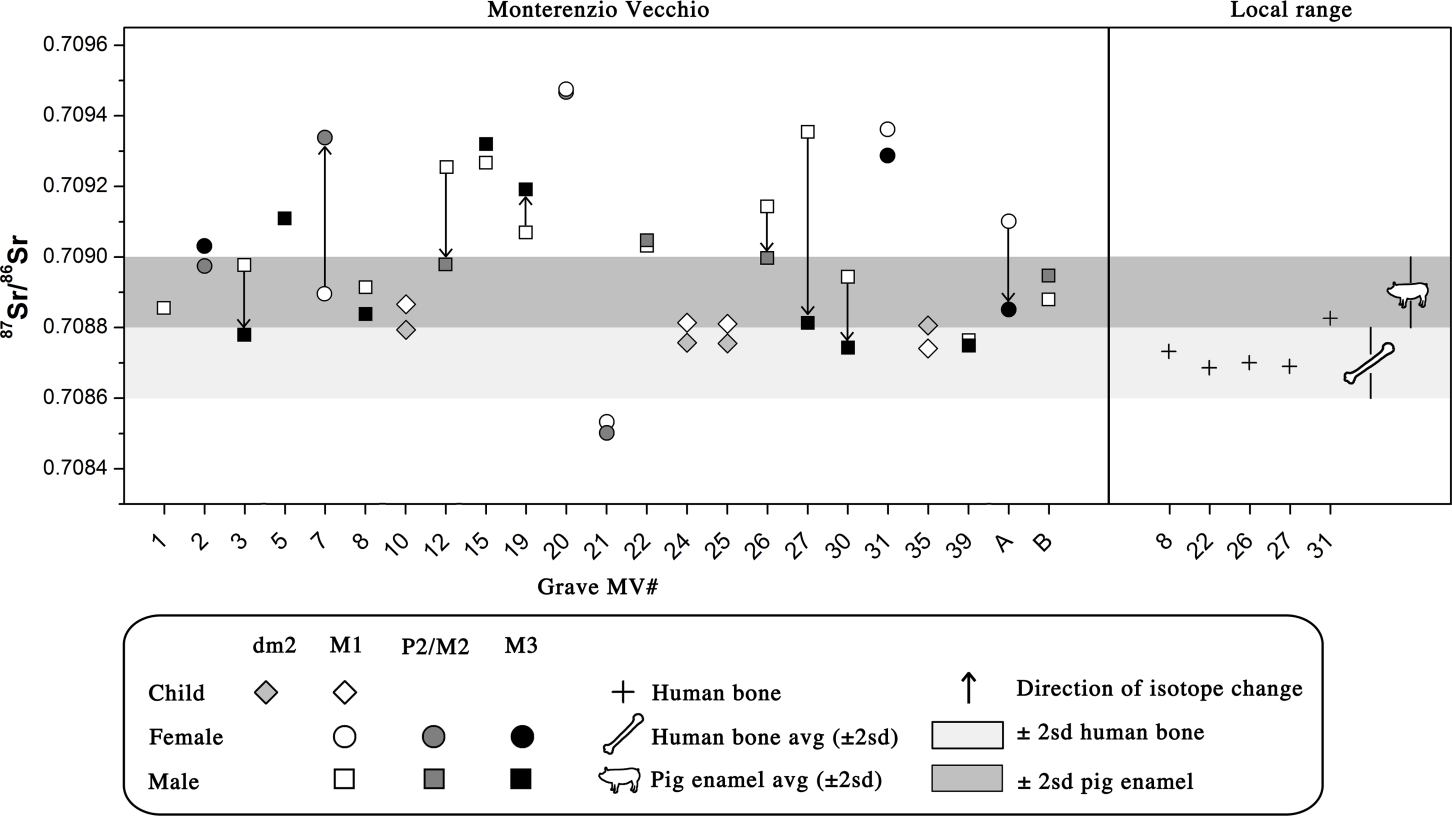
Strontium isotope results on provenance and mobility patterns of individuals of Monterenzio Vecchio. Pig enamel data are from Scheeres et al. (2013).

The local Sr isotope signature of Monterenzio Vecchio has been determined by analyzing five human bones (MV 8, 22, 26, 27, 31), obtaining a mean ^87^Sr/^86^Sr ratio of 0.7087 ± 0.0001 (2σ). This value is very similar, even if slightly lower, to the previously reported bioavailable Sr isotopic ratio for the Monterenzio Vecchio site by Scheeres et al., 2013 [19] (0.7089 ± 0.0001 [2σ]) obtained from the analysis of five pig enamel samples. The results of our human bones and the pig enamel of Scheeres et al., 2013 [19] do not overlap, but they are especially close to each other in terms of the Sr isotopic range. Moreover, diet and amino acid requirements of pigs are similar to those of humans and, therefore, pigs can be used as a model to construct the local Sr baseline [98]. The difference in the two intervals might be related to a different diagenetic uptake usually more severe for bone tissue. Thus, we choose to consider the broadest possible ^87^Sr/^86^Sr interval as the local bio-available strontium isotopic ratio, combining our results with those of Scheeres et al., 2013 [19], to avoid an overestimation of the non-local individual number. Therefore, the resulting local range used in this work falls between 0.7090 and 0.7086 (Fig 2). This interval is also compatible with the Sr isotopic signature of Miocene-Pleistocene calcareous rocks outcropping in the area, derived from the interpretation of the Sr isotope seawater curve of McArthur et al., 2001 [99], broadly exploited to date calcareous marine rocks throughout the Tertiary.

Further to this, our samples of human enamel from the Monterenzio individuals yielded ^87^Sr/^86^Sr ratios ranging between 0.7095 and 0.7085, with a mean ^87^Sr/^86^Sr ratio of 0.7090 ± 0.0005 (2σ). The broad Sr isotope range of the individuals with respect to the local Sr isotope signature (Table 2, Fig 2) suggests the presence of local and non-local isotopic signatures in the Monterenzio Vecchio necropolis. The ^87^Sr/^86^Sr values obtained from the teeth mineralized during early childhood and intrauterine life, the M1s of seven adults (six males and one female) and dm2s of four subadults fall within the local isotope range. This might mean that the mother of these individuals was living in Monterenzio Vecchio during pregnancy and that the individuals likely were born and lived at least a few of their early childhood years in Monterenzio Vecchio or a geologically similar location. By contrast, the ^87^Sr/^86^Sr ratios for M1s of 10 other individuals (six males and four females) vary between 0.7090 and 0.7096, falling outside the local range. This might imply a non-local provenance. Moreover, one individual (MV21) has a low M_1_ Sr isotope ratio (= 0.7085), outside both the local and non-local main ranges of Monterenzio, likely indicative of an additional non-local signature. For two individuals (MV2 and MV5) the M^2^ (childhood) and M_3_ (late childhood) were sampled but not M1. The M^2^ for individual MV2 (female) shows local values, whereas the M_3_ for individual MV5 (male) shows non-local values. The non-local ^87^Sr/^86^Sr ratios, ranging between 0.7090 and 0.7096, are comparable with those reported by Scheeres et al., 2013 [19] for non-local individuals of Monte Bibele (0.7092–0.7094). Accordingly, the Sr isotope composition of childhood teeth reveals that 11 individuals out of the 23 analyzed had likely spent their childhood non-locally.

Intra-individual patterns of mobility were investigated in 21 humans using the enamel of two teeth with different mineralization age. Four subadults (MV 10, 24, 25, 35) and five adult males (MV 30, B, 39, 8, 3) have similar ^87^Sr/^86^Sr values in both of their teeth, indicating a limited mobility during childhood and a likely local origin. In contrast, three males (MV 19, 15, 22) and three females (MV 31, 20 and 21) show non-local Sr isotope values in both of their teeth, suggesting that they arrived in Monterenzio Vecchio at some point during childhood. Moreover, individual MV21 has likely arrived to Monterenzio from a different locality with respect to the majority of the non-locals. Furthermore, four individuals (three males and a female, respectively MV 27, 26, 12 and MV A) show different Sr ratios in their teeth, reflecting movements in late childhood from a place of origin with high Sr isotopes ratios to a place with lower Sr isotopes that we identify as Monterenzio. Interestingly, two females (MV 2 and more conspiciously MV7) show an inverse trend of mobility, suggesting they moved from a low Sr isotope (as Monterenzio) to a high Sr isotope ratio end-member during their childhood and then likely returned to the original low Sr ratio locality later on.

### Non-metric dental traits

Frequencies of 14 traits for 19 samples are listed in Table 4. Following the isotope results, the individuals of Monterenzio Vecchio (MV) were divided in two groups: local (MVL) and non-locals (MVNL). MVL includes eight individuals (MV 1, 2, 3, 7, 8, 30, 39 and B) and MVNL groups 10 individuals (MV 5, 12, 15, 19, 20, 21, 22, 26, 27 and A). As the isotope results are not available for individual MV 36, this individual was only included in the analysis that considers the whole sample (i.e., MV).

**Fig 3:**
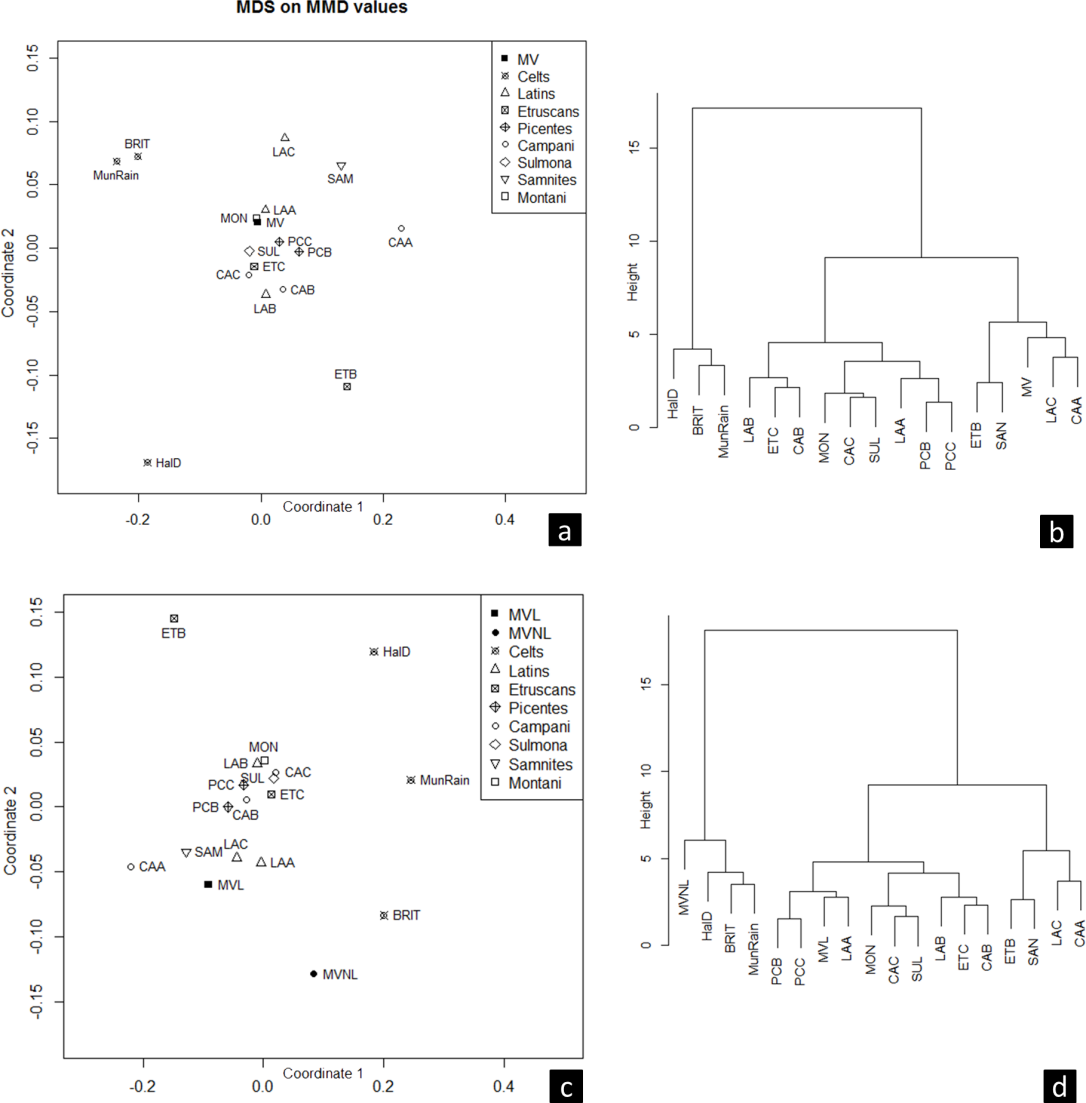
Two-dimensional MDS and cluster dendrogram based on MMD values for Italics and Celts: (a, b) Monterenzio Vecchio (MV; pooled) and (c, d) Monterenzio Vecchio locals (MVL) and non-locals (MVNL), respectively.

Since the M_1_ deflecting wrinkle and the M_1_ trigonid crest have few observations and are strongly underestimated in MV (mainly due to excessive dental wear), they were removed from further considerations. For the remaining 12 traits in the MV sample, the Pearson chi square test was carried out to remove correlated traits. The I^2^ interruption groove and M_1_ protostylid were thus removed from further analyses because they present higher correlations (I^2^ interruption groove / M^1^ Carabelli's trait, r = 0.63; I^2^ interruption groove / P^1^ premolar root number, r = 0.56; I^2^ interruption groove / M_1_ protostylid, r = 0.78; M_1_ protostylid / I^2^ tuberculum dentale, r = 0.53; M_1_ protostylid / M^1^ Carabelli's trait, r = 0.73). In sum, 10 of the 14 non-metric dental traits were used for MMD analysis (Tables 4 and 5), which in turn were used for two-dimensional MDS and cluster analysis (Fig 3).

**Table 5:** MMD value (below) and SD (above) for 10 dental traits among Monterenzio Vecchio, MV locals and non-locals, Celtic and Italian Iron Age samples^1^.

	MV	MVL	MVNL	BRIT	MunRain	HalD	LAA	LAB	LAC	ETB	ETC	PCB	PCC	CAA	CAB	CAC	SUL	SAM	MON
MV		0.109	0.097	0.046	0.045	0.047	0.078	0.08	0.101	0.066	0.054	0.042	0.038	0.055	0.077	0.053	0.064	0.042	0.059
MVL	-0.123		0.14	0.091	0.09	0.092	0.123	0.124	0.14	0.11	0.099	0.087	0.083	0.1	0.123	0.098	0.108	0.087	0.103
MVNL	-0.138	0.005		0.079	0.078	0.079	0.109	0.11	0.125	0.097	0.086	0.074	0.071	0.087	0.108	0.085	0.096	0.074	0.09
BRIT	**0.14**	**0.223**	0.056		0.028	0.029	0.061	0.064	0.089	0.05	0.037	0.024	0.021	0.038	0.059	0.035	0.046	0.024	0.042
MunRain	**0.306**	**0.409**	**0.222**	0.05		0.028	0.06	0.063	0.089	0.049	0.036	0.024	0.02	0.037	0.059	0.034	0.046	0.023	0.041
HalD	**0.241**	**0.299**	**0.207**	**0.142**	**0.245**		0.061	0.064	0.089	0.05	0.037	0.025	0.021	0.038	0.06	0.036	0.047	0.024	0.042
LAA	-0.009	-0.011	-0.026	0.108	**0.225**	**0.223**		0.096	0.118	0.082	0.069	0.057	0.053	0.07	0.091	0.068	0.078	0.056	0.074
LAB	0.1	-0.025	0.186	**0.151**	**0.248**	0.086	-0.036		0.116	0.086	0.073	0.06	0.056	0.073	0.094	0.07	0.081	0.059	0.077
LAC	0.165	0.046	**0.254**	**0.204**	**0.265**	**0.329**	0.062	0.004		0.103	0.093	0.086	0.084	0.096	0.11	0.096	0.099	0.086	0.097
ETB	**0.231**	0.084	**0.348**	**0.409**	**0.391**	**0.284**	0.127	-0.07	0.19		0.059	0.046	0.043	0.059	0.081	0.056	0.067	0.046	0.064
ETC	0.027	-0.018	0.06	**0.135**	**0.173**	**0.126**	-0.026	-0.09	0.088	-0.029		0.033	0.03	0.046	0.069	0.044	0.055	0.033	0.051
PCB	**0.119**	0.056	0.142	**0.254**	**0.283**	**0.27**	-0.029	-0.041	0.121	0.005	-0.022		0.016	0.033	0.056	0.031	0.042	0.02	0.038
PCC	**0.143**	0.018	**0.207**	**0.201**	**0.249**	**0.24**	0.019	-0.052	0.114	0.071	0.004	0.02		0.03	0.052	0.027	0.039	0.016	0.034
CAA	**0.221**	0.116	**0.253**	**0.405**	**0.484**	**0.435**	0.062	0.025	0.058	0.108	**0.097**	0.013	**0.1**		0.069	0.044	0.055	0.033	0.051
CAB	-0.003	-0.135	0.076	**0.191**	**0.291**	**0.198**	-0.024	-0.095	0.137	-0.015	-0.067	-0.037	-0.064	0.065		0.066	0.077	0.055	0.073
CAC	0.068	0.05	0.092	**0.159**	**0.151**	**0.133**	0.048	-0.048	0.106	0.035	-0.059	0.039	0.042	**0.154**	-0.013		0.052	0.031	0.048
SUL	0.126	0.056	0.157	0.09	**0.169**	**0.188**	0.095	-0.048	0.142	0.012	-0.028	0.051	0.061	**0.168**	-0.011	0.043		0.042	0.059
SAM	**0.135**	-0.027	**0.225**	**0.306**	**0.361**	**0.41**	0.089	-0.004	**0.175**	0.051	0.021	**0.045**	0.017	**0.113**	-0.067	0.06	0.045		0.037
MON	0.107	-0.032	**0.223**	**0.158**	**0.169**	**0.226**	0.05	-0.073	0.038	-0.008	-0.042	0.049	0.013	**0.191**	-0.038	-0.001	-0.032	0.022	

^1^Bold values indicate significant distances at the 0.025 level.

As shown in the MDS plot (Fig 3A), the Celts separate from the Italics (with a MMD range of 0.126–0.484), the former falling on the left side of the plot, while the Italics are grouped in a central position. The MV sample falls within the Italic variability, and specifically closer to LAA (MMD = -0.009) and CAA (MMD = -0.003) (Table 5). Similar results are obtained by the Cluster dendrogram (Fig 3B), which shows two main clusters (i.e., the Celts and the Italics), with MV included within the Italics. The separation between Celts and Italics is mainly driven by the M^1^ Carabelli's trait and the M^2^ molar root number, both reflecting lower frequencies among the Celts (Table 4).

A different scenario is observed when the individuals of Monterenzio Vecchio are grouped based on the isotope results (i.e., MVL and MVNL). Both MDS (Fig 3C) and hierarchical cluster analysis (Fig 3D) separate MVNL, which plots and cluster close to the Celts (in particular to BRIT with a MMD value of 0.056), from MVL, which instead plots within the Italics (mean MMD 0.013). The MVL sample is characterized by relatively higher frequencies of M^1^ Carabelli's trait, P^1^ premolar root number and lower incidence of I^1^ double shovelling and M^3^ parastyle than MVNL.

### Archaeological data

**Fig 4:**
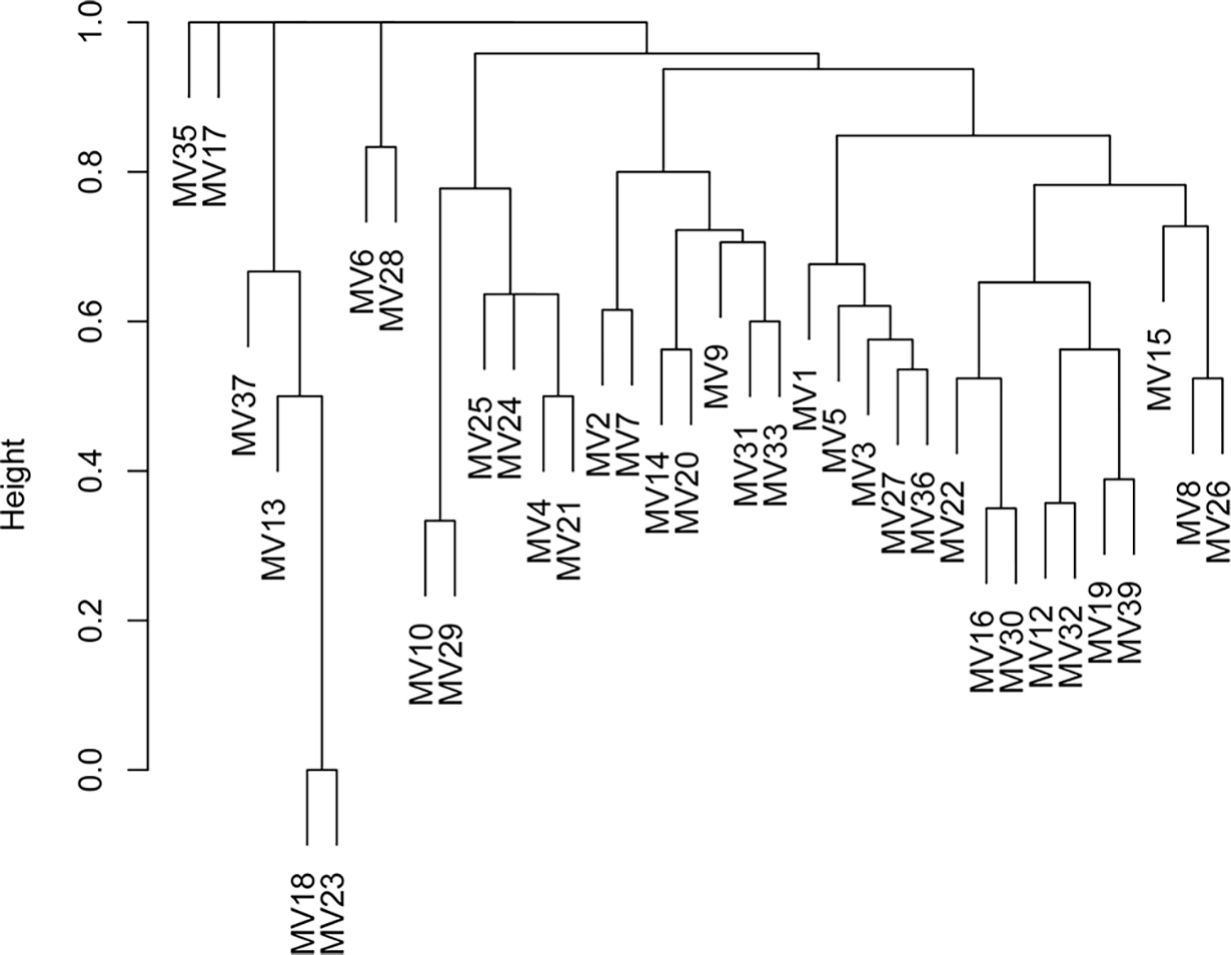
Complete linkage hierarchical cluster of all individuals. The analysis is based on pairwise Jaccard distances computed based on presence-absence values of funerary items as indicated in Table 5.

We visually assess the impact of ethnic origin/background on the distribution of grave goods and compare this effect with the impact of other variables known from anthropological analysis (i.e., sex and age) through hierarchical clustering. The resulting plot (Fig 4) an inconsequential relationship of ethnic background in determining population structure. The graph exhibits instead clear segregation patterns due to sexual dimorphism and different age cohorts (S1 Text), with a broad tendency of females to cluster with infants and children on the left side of the graph, and males tending to cluster together on the opposite side. The four cremations, for which the sex is unknown, plot both in the male (MV 16 and 32) and in the female (MV 6 and 28) clusters. The impact of sexual dimorphism is statistically significant (χ^2^ = 130.14; df = 64; p<0.001). Conversely, difference in age or origin do not show significant differences (χ^2^ = 323.86, df = 325, p-value = 0.5; χ^2^ = 94.04; df = 132, p-value = 0.993 respectively; S2 Text, S1 Fig, S2 Table), and the majority of grave goods is almost uniformly distributed across provenance classes based on Sr isotope values (S2 Text, S3 Table). These results are confirmed by AMOVA (S2 Text), which yielded high and significant values for sex and age (Φ_ST_ = 0.16, p<0.001; Φ_ST_ = 0.12, p<0.001 respectively), but no significant population structure due to origin/ethnic background (Φ_ST_ = -0.01, p = 0.72).

To identify the cultural variables that are more likely to be linked to sexual dimorphism in the present dataset we use a Random Forest classification algorithm on a subset comprising only 21 adult individuals (S4 Table). The results appear to robustly identify (90% accuracy, 9.52% error rate for Out of Bagging data; see S3 Text) a number of cultural markers that could be directly linked to sex based on their Gini index (i.e., whorls, skyphos, mirror, different types of pearls to females, while iron sword with scabbard, iron knife, kylix, iron javelin, iron spear, mortar, shear, strigil, razor and many others to males; S2 Fig). If the variables that have a stronger impact on supervised classification based on sex are not considered (i.e., higher than 0.1 Gini coefficient; S4 Table), Random Forest yields a ranking of variables with more noise (47% accuracy, 52.92% error rate in Out of Bagging data; S3 Text, S3 Fig, S5 Table) which are more likely to be linked to differential origin. For example, stemmed plate, ceramic kyathos, and bowl are more often associated with non-local individuals, while kantharos, fibula, jar, amber pearl, pendant, helmet, and other traits could be associated with local origin.

## Discussion

Archaeological data suggest that the arrival of the Celts in the Po Valley during the 4^th^ and 3^rd^ centuries BC led to different patterns of interaction with the indigenous communities [1,2,5]. The funerary remains linked to Celtic presence in the area of Bologna are of great importance for understanding the interaction patterns developed between Etruscans and Celts. On one hand, funerary contexts suggest the presence of conflict or competition between the two ethnic groups (e.g., the necropolis of Casalecchio di Reno "Zone A" and the site of Marzabotto) [4,10–13]. On the other hand, comparable and coeval funerary contexts have also demonstrated a gradual process of integration, where Celts adopted the Etruscan lifestyle while Etruscans absorbed Celtic funerary ideology and began to place weapons in their graves (e.g., the necropolis of Felsina, the necropolis of Monte Bibele and the necropolis of Monterenzio Vecchio) [3,5–7,13]. Nonetheless, cultural assemblages do not always form a reliable proxy of population turnover, as cultural change may be driven by plain cultural diffusion without necessarily implying the arrival of newcomers [55,57,100–103]. Paradoxically, up to now, there was no biological evidence to confirm the immigration of Celts in northern Italy, and a more comprehensive biocultural approach was never used to address issues of admixture between these two populations. Within this context, Monterenzio Vecchio is the first archaeological site in northern Italy to be investigated for understanding the biocultural relationship between Etruscans and Celts.

The results based on strontium isotopes likely indicate that more than half of the adults in Monterenzio Vecchio (58%) are non-local, a much higher proportion than that observed by Scheeres et al., 2013 [19] at the nearby site of Monte Bibele (19%). As in Monte Bibele, all non-local individuals analyzed in Monterenzio Veccho exhibit Sr isotopic ranges above 0.7090. Interestingly, Sr isotope values also suggest that some individuals moved to Monterenzio Vecchio during their childhood, while some others later on in life. Moreover, individuals MV7 and MV2 show an inverse pattern, i.e., from Monterenzio Vecchio to outside regions. Nevertheless, isotope results cannot inform us whether allochtnous individuals are of possible Celtic origin, or if they rather belong to other, non-local Italian groups.

To resolve this issue we used non-metric dental traits, which are strongly linked to genetic background and are therefore suitable to highlight biological relationships between populations (e.g., [29,35,41,43–46]). To our knowledge, only Thompson et al., 2015 [104] combined Sr isotopes and non-metric dental traits to identify potential biological relatedness and population movement in past societies. However, in our contribution, for the first time both analyses were undertaken on the very same individuals. Therefore, the two groups identified by Sr isotope analysis (Monterenzio Vecchio Local: MVL; Monterenzio Vecchio non-local: MVNL) were considered to compute inter-group biological divergence based on the analysis of non-metric dental traits (i.e., MMD), ultimately providing a unique opportunity to formally assess closeness between non-local individuals (i.e., MVNL) and other groups. Interestingly, the MVL group diverges from the Celts and aligns with the Italic sample. Conversely, the MVNL group is closer to the Celts, in particular with non-continental Celts from Yorkshire (BRIT). We do not have an explanation for why there is a higher biological relatedness with BRIT Celts, yet our results attests that Celtic groups effectively arrived in Monterenzio Vecchio during the 4^th^ and 3^rd^ centuries BC. For most adult individuals in this sample, Sr isotope analysis was undertaken on the enamel of the M1s which tend to mineralize from < 5 months to 5.5 years of age [105]. These results suggest that young individuals actively took part in the migration, supporting the hypothesis that the Celtic migration was an event that involved entire communities [13,21,61].

A qualitative and quantitative exploration of variability in grave goods has shown that ethnic origin/background has negligible impact on the distribution of funerary elements. By contrast, differences between sex have a much stronger effect on population structure, followed by marked differences due to age class, in particular between infant/children on the one hand, and adults on the other. All the analyses, however, confirmed the relevance of weaponry in discriminating between males of autochthonous and allochthonous origin.

## Conclusions

In sum, Monterenzio Vecchio emerges as a critical case study for exploring demic processes of cultural transmission in past populations, with particular reference to possible relationships between population movement/replacement and the degree of cultural admixture that takes place between demes. The context allows us to observe independent archaeological and anthropological evidence in a framework of historically documented migration of Celtic populations into a circumscribed area of northern Italy. The comparison of a wide range of markers testifies to events of human movement possibly followed by a high degree of cultural admixture between exogenous and endogenous traits. This resulted in a form of cultural syncretism, which is masking most of the variability due to provenance or cultural background, while anthropological markers still show a clearer segregation between individuals with local ancestry and individuals with non-local origin. In this context of increasing cultural homogeneity, between the end of the 4^th^ and the end of the 3^rd^ century BC, the population of Monterenzio Vecchio used other means to convey interpersonal diversity in funerary practices, such as sex of the individual and markers of age—in particular, discriminating between infants and adults.

Considering all evidence produced across the different and yet complementary array of archaeological and anthropological results, the present study sets an example for the exploration of biocultural processes in past and present populations, and testifies to the many differences in mode and time that may exists between population replacement and processes of cultural admixture at a local scale. Future research will focus on the analysis of other Iron age samples in the territory of Bologna where different Celtic groups arrived in different period as is attested in the "Zone A" at Casalecchio di Reno that refers to the first migration of such populations in these areas without apparent mixing with locals. Ultimately, the study of the markers of biodistance may provide new insight on the migration and interactions of populations in the Po valley.

## Supporting information

S1 Text Cluster analysis. (PDF)

S2 Text Correspondence analysis and measures of association. (PDF)

S3 Text Classification and estimate of variable relevance through Random Forest. (PDF)

S1 Fig Correspondence analysis plot based on age classes. C, Child; YA, Young Adult; A, Adult; MA, Middle Adult; OA, Old Adult; X, unknown sex.
(PDF)

S2 Fig Dot chart indicating the decay in variable importance for classification based on sex measured as Gini coefficient through Random Forest. (PDF)

S3 Fig Dot chart indicating the decay in variable importance for classification based on origin measured as Gini coefficient through Random Forest on sex-unbiased, non collinear variables. (PDF)

S1 Table Grave goods at Monterenzio Vecchio used in analysis of archaeological data. ^1^Origins based on isotopic results: 0 = local, 1 = missing data, 2 = non local.
(PDF)

S2 Table Correspondence analysis on age classes. (PDF)

S3 Table Correspondence analysis on provenance. (PDF)

S4 Table Subset of 21 adult individuals used in Random Forest analysis. (PDF)

S5 Table Subset of 17 adult individuals and 41 uncorrelated variables used in Random Forest analysis. (PDF)

S6 Table Confusion matrix for sex (OOB error = 9.52%). (PDF)

S7 Table Confusion matrix for origin (OOB error = 52.94%). (PDF)
