# Supplementary material for: Unravelling biocultural population structure in 4th/3rd century BC Monterenzio Vecchio (Bologna, Italy) through a comparative analysis of strontium isotopes, non-metric dental evidence, and funerary practices
Source: PLoS One. 2018 Mar 28;13(3):e0193796. doi: 10.1371/journal.pone.0193796 (PMC5874009; doi:10.1371/journal.pone.0193796)
Supplement: S1 Text — (PDF) [file pone.0193796.s001.pdf]

S1 Text. Cluster analysis

Hierarchical clustering (Fig 4), computed on all individuals based on Jaccard distance shows that the possible structure exhibited by this population does not mirror ethnic background or provenance. The resulting dendrogram shows instead a clear segregation between Infants and Children on one side (the most basal branches of the tree) and another, more conspicuous, cluster in itself divided between Females and Males. It therefore appears that differences between adults and children are one of the main drivers of variability in grave goods, followed by sexual dimorphism among adults.
