# Supplementary material for: Unravelling biocultural population structure in 4th/3rd century BC Monterenzio Vecchio (Bologna, Italy) through a comparative analysis of strontium isotopes, non-metric dental evidence, and funerary practices
Source: PLoS One. 2018 Mar 28;13(3):e0193796. doi: 10.1371/journal.pone.0193796 (PMC5874009; doi:10.1371/journal.pone.0193796)
Supplement: S2 Text — (PDF) [file pone.0193796.s002.pdf]

S2 Text. Correspondence Analysis and measures of association

A Chi-squared test confirms the significant sexual dimorphism existing at Monterenzio Vecchio (chi-square = 130.14; df = 64; p<0.001). It is apparent that some items are exclusively associated with one sex. As far as male individuals are concerned, this is true for weapons (iron sword with scabbard, iron javelin, iron spear, shield, iron knife), items for personal care (iron shear, razor, strigil), valuable tokens (bronze belt rings), proto currency (aes rude), and specific vessel types (mortar, painted cup, bronze kyathoi, bronze colum). Females are instead preferentially associated with personal ornaments (mirror, pearls, iron bracelet), items for specific activities (whorls), and different vessel types (skyphos). Fibula and plate (bucchero) are widespread across sexes.

In relation to age cohorts (S2 Table), the first two axes of Correspondence Analysis explain approximately 63.4% of the total variability in the dataset (S1 Fig). The analysis suggests that Axis 1 divides between Young Adults, Adults, and Mature Adults - which tend to cluster together - on the one hand, and Infants, Children and Old Adults on the other. Further differentiation between Infants and elderly individuals is described by Axis 2, where these two groups fall on opposite extremes of the spectrum. A closer inspection reveals that this intergroup difference is mostly due to the fact that miniaturized vase and globular vase (figuline) are preferentially found with Infants and Children, while large vessels in a variety of materials are preferentially attributed to Old Adults (skyphos, pitcher, krater, situla) as well as other ceramic forms (black-glazed plate) and personal ornaments (e.g., mirror, sea shells). Weapons, on the other hand, strongly characterise the male group comprising Young Adults, Adults, and Mature Adults (iron sword with scabbard, iron javelin, iron spear, shield, iron knife. Bowl (bucchero) and fibulae are almost equally present in all age classes.

Finally, a Correspondence Analysis run on provenance groups is able to describe all the observed variability in grave good using only the first two principal axes (S3 Table). There are attributes exhibited only by non-local individuals (cereal seeds, sea shells, iron cylinder, whetstone, iron hoe, trefoil oinochoe, jar-bucchero). On the other hand, local individuals preferentially have kelébe, personal ornaments (pearls, pendants), that may be suggestive of many local individuals being female. The majority of grave goods are almost uniformly distributed across provenance classes as testified by AMOVA and a chi square test (chi-square = 94.04, df = 132, p-value = 0.993). However, there are a few traits that are not exhibited by undetermined individuals, such as kantharos, stamens jar, cup (bucchero), black-glazed plate, mirror, iron bracelet), which may be interesting - as well as other elements exhibited only by undetermined individuals (bone pearl, bronze leaf, firedog).
