# Supplementary material for: Unravelling biocultural population structure in 4th/3rd century BC Monterenzio Vecchio (Bologna, Italy) through a comparative analysis of strontium isotopes, non-metric dental evidence, and funerary practices
Source: PLoS One. 2018 Mar 28;13(3):e0193796. doi: 10.1371/journal.pone.0193796 (PMC5874009; doi:10.1371/journal.pone.0193796)
Supplement: S3 Text — (PDF) [file pone.0193796.s003.pdf]

S3 Text. Classification and estimate of variable relevance through Random Forest

The possible relationship between individual variables and grouping of the studied individuals into pre-arranged categories (i.e., Male/Female, and Local/non-Local provenance) was assessed via a Random Forest classification algorithm. The first experiment on sexual dimorphism yielded a clear ranking of variables (S2 Fig) accompanied by an accurate classification prediction based on the distribution of grave goods. The resulting confusion matrix reported in S6 Table shows that – based on cultural attributes – individuals at Monterenzio Vecchio were misattributed to males (1) or females (0) only once per each category, therefore giving a quite high accuracy (19 correct cases of 21 total cases = 90%). In addition, the validation against Out of Bagging data (OOB) resulted in a very limited error rate (9.52%). The highest-ranking variables based on their mean decrease in Gini coefficient (S2 Fig; from whorls to ring) were then taken out of the dataset, and the remaining variables were used to perform a second Random Forest, this time aimed at identifying characters that were more likely linked to a local (0) or non-local (1) origin of Monterenzio individuals. This second experiment yielded a ranking of objects that may be linked to a different ethnic background (S3 Fig). However, there results are less reliable than the previous ones. The second confusion matrix (S7 Table) shows that cases of misattribution are almost as common as correct classifications for local individuals, and more common than correct attributions for non-local ones. The final accuracy of the analysis is therefore equal to 47%, with 8 correct cases out of 17 total cases.
