# Supplementary material for: Unravelling biocultural population structure in 4th/3rd century BC Monterenzio Vecchio (Bologna, Italy) through a comparative analysis of strontium isotopes, non-metric dental evidence, and funerary practices
Source: PLoS One. 2018 Mar 28;13(3):e0193796. doi: 10.1371/journal.pone.0193796 (PMC5874009; doi:10.1371/journal.pone.0193796)
Supplement: S2 Fig — (PDF) [file pone.0193796.s005.pdf]

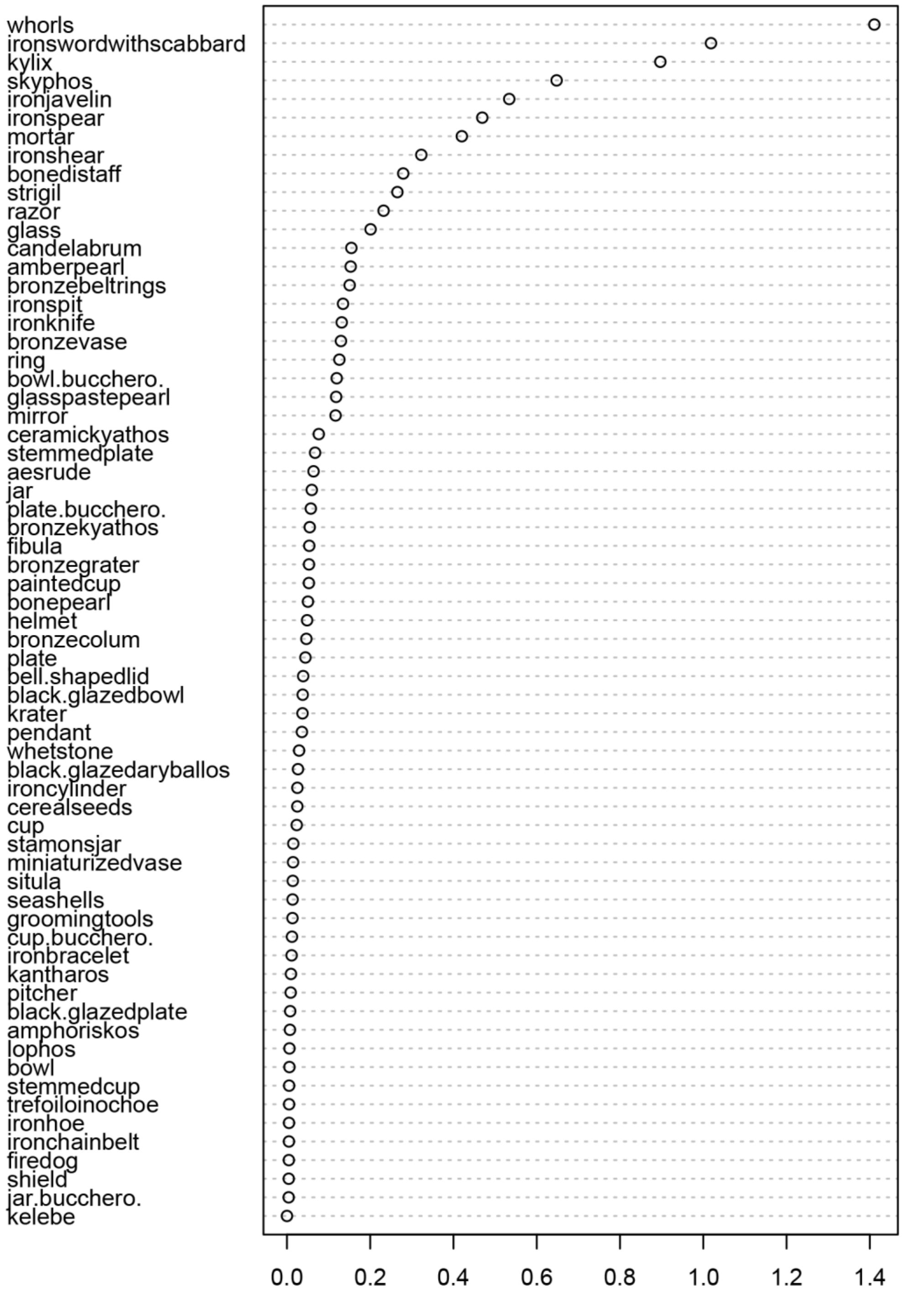

S1 Fig. Dot chart indicating the decay in variable importance for classification based on sex measured as Gini coefficient through Random Forest.
