# Supplementary material for: Unravelling biocultural population structure in 4th/3rd century BC Monterenzio Vecchio (Bologna, Italy) through a comparative analysis of strontium isotopes, non-metric dental evidence, and funerary practices
Source: PLoS One. 2018 Mar 28;13(3):e0193796. doi: 10.1371/journal.pone.0193796 (PMC5874009; doi:10.1371/journal.pone.0193796)
Supplement: S3 Fig — (PDF) [file pone.0193796.s006.pdf]

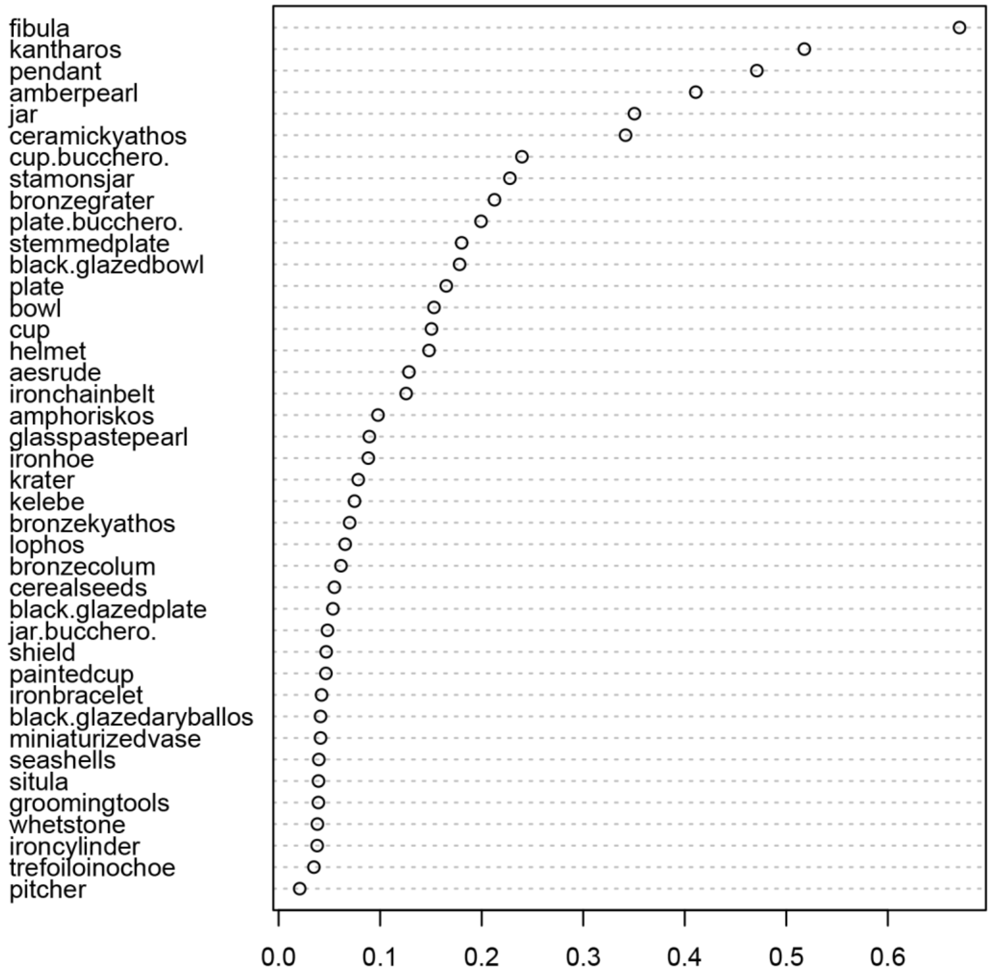


**S3 Fig. Dot chart indicating the decay in variable importance for classification based on origin measured as Gini coefficient through Random Forest on sex-unbiased, non collinear variables.**
