# Supplementary material for: Unravelling biocultural population structure in 4th/3rd century BC Monterenzio Vecchio (Bologna, Italy) through a comparative analysis of strontium isotopes, non-metric dental evidence, and funerary practices
Source: PLoS One. 2018 Mar 28;13(3):e0193796. doi: 10.1371/journal.pone.0193796 (PMC5874009; doi:10.1371/journal.pone.0193796)
Supplement: S1 Table — 1Origins based on isotopic results: 0 = local, 1 = missing data, 2 = non local. (PDF) [file pone.0193796.s007.pdf]

S1 Table. Grave goods at Monterenzio Vecchio used in analysis of archaeological data.

		Individuals																																			
Variables	MV	1	2	3	4	5	6	7	8	9	10	12	13	14	15	16	17	18	19	20	21	22	23	24	25	26	27	28	29	30	31	32	33	35	36	37	39
origin[1]		0	0	0	1	2	1	0	0	1	0	2	1	1	2	1	1	1	2	2	2	2	1	0	0	2	2	1	1	0	2	1	1	0	1	1	0
Skyphos		0	1	0	1	1	0	1	0	1	0	0	0	1	0	0	0	0	0	1	0	1	0	1	0	0	0	0	0	0	1	0	1	0	0	0	0
trefoil oenochoe		0	1	0	0	0	0	0	0	0	0	0	0	0	0	0	0	0	0	0	0	0	0	0	0	0	0	0	0	0	0	0	0	0	0	0	0
Kylix		1	0	1	0	1	0	0	1	0	0	1	0	0	1	1	0	0	1	0	1	0	0	0	0	1	1	0	0	1	0	1	0	0	1	0	1
Mortar		1	0	1	0	1	0	0	0	0	0	1	0	0	1	1	0	0	1	0	0	0	0	0	0	0	1	0	0	1	0	1	0	0	0	0	0
Pitcher		0	0	0	0	1	0	0	0	0	0	0	0	0	0	1	0	0	0	0	0	0	0	0	0	0	0	0	0	0	0	0	0	0	0	0	0
Kelébe		0	0	1	0	0	0	0	0	0	0	0	0	0	0	0	0	0	0	0	0	0	0	0	0	0	0	0	0	0	0	0	0	0	0	0	0
Kantharos		1	1	0	0	0	0	1	0	0	0	0	0	0	0	0	0	0	0	0	0	0	0	0	0	0	0	0	0	0	0	0	0	0	0	0	0
ceramic kyathos		0	0	0	0	0	0	0	1	1	0	0	0	0	0	1	0	0	1	0	1	0	0	0	0	1	0	0	0	1	0	0	0	0	0	0	0
Krater		0	0	0	0	0	0	1	0	0	0	0	0	1	0	0	0	0	0	0	0	0	0	0	0	0	0	0	0	0	0	0	0	0	1	0	0
globular vase (figuline)		0	0	0	0	0	0	0	0	0	0	0	0	0	0	0	0	0	0	0	0	0	0	1	0	0	0	0	0	0	0	0	0	0	0	0	0
jar (bucchero)		0	0	0	0	0	0	0	0	0	0	0	0	0	0	0	0	0	0	0	0	0	0	0	0	0	1	0	0	0	0	0	0	0	0	0	0
stamons jar		0	0	1	0	0	0	0	1	0	0	0	0	0	0	0	0	0	0	0	0	0	0	0	0	0	0	0	0	0	1	0	0	0	0	0	0
Jar		1	1	1	1	0	1	0	1	1	0	1	0	0	1	1	0	0	1	1	1	1	0	0	1	0	0	1	0	1	0	1	0	0	1	0	1
black-glazed bowl		1	1	1	0	1	1	1	0	0	0	0	0	0	0	0	0	0	0	0	0	0	0	0	0	1	1	0	0	0	0	0	1	0	1	0	0
Bowl		0	0	1	1	1	0	1	1	1	1	1	0	1	1	1	0	0	1	1	1	1	0	1	1	1	1	0	1	1	1	1	1	0	1	0	1
bowl (bucchero)		0	0	0	0	1	0	0	1	0	0	1	0	1	1	1	1	0	1	1	1	1	0	1	1	1	1	0	0	1	1	1	1	0	1	0	1
stemmed cup		0	0	0	0	0	0	0	0	0	0	0	0	0	0	0	0	0	0	0	0	0	0	0	0	0	0	0	0	0	0	0	0	0	1	0	0
painted cup		1	0	0	0	1	0	0	0	0	0	0	0	0	0	1	0	0	0	0	0	0	0	0	0	0	0	0	0	0	0	0	0	0	1	0	0
Cup		1	0	0	0	1	0	1	0	0	0	0	0	0	0	0	0	0	0	0	0	0	0	0	0	0	0	0	0	0	1	0	0	0	0	0	0
cup (bucchero)		1	1	0	0	0	0	0	0	0	0	0	0	0	0	0	0	0	0	0	0	0	0	0	0	0	0	0	0	0	0	0	0	1	0	0	0
black-glazed plate		0	0	0	0	1	0	1	0	0	0	0	0	0	0	0	0	0	0	0	0	0	0	0	0	0	0	0	0	0	0	0	0	0	0	0	0
stemmed plate		1	1	0	0	1	0	0	0	0	0	0	0	1	1	1	0	0	0	1	0	1	0	0	0	0	1	0	0	1	0	0	0	0	1	0	0
plate (bucchero)		1	1	0	1	0	0	0	1	1	0	1	0	0	0	0	0	0	1	0	1	0	0	1	1	1	0	0	0	0	1	1	0	1	0	0	1
Plate		0	1	1	0	0	0	0	1	1	0	0	0	0	0	0	0	0	1	0	0	0	0	0	0	1	0	0	0	0	1	0	1	0	0	0	0
Glass		1	1	1	1	1	0	1	1	0	1	1	0	1	1	1	0	0	1	0	1	1	0	1	1	1	0	0	1	1	0	1	1	0	1	0	1
miniaturized vase		0	1	0	0	0	0	0	0	0	0	0	1	0	0	1	0	1	0	0	0	0	1	0	0	0	0	0	0	0	0	0	0	0	0	1	0
bronze kyathos		0	0	1	0	1	0	0	0	0	0	0	0	0	0	0	0	0	0	0	0	0	0	0	0	0	0	0	0	0	0	0	0	0	1	0	0
Situla		0	0	0	0	1	0	0	0	0	0	0	0	0	0	0	0	0	0	0	0	0	0	0	0	0	0	0	0	0	0	0	0	0	0	0	0
iron sword with scabbard		1	0	1	0	0	0	0	0	0	0	1	0	0	0	1	0	0	1	0	0	1	0	0	0	1	1	1	0	1	0	1	0	0	1	0	1
bronze belt rings		1	0	0	0	0	0	0	0	0	0	0	0	0	0	1	0	0	1	0	0	1	0	0	0	1	0	0	0	0	0	1	0	0	0	0	0
iron chain belt		0	0	1	0	0	0	0	0	0	0	0	0	0	0	0	0	0	0	0	0	0	0	0	0	0	0	0	0	0	0	0	0	0	0	0	0
iron javelin		1	0	1	0	0	1	0	0	0	0	1	0	0	0	1	0	0	1	0	0	1	0	0	0	1	1	1	0	1	0	1	0	0	0	0	0
iron spear		1	0	0	0	0	0	0	0	0	0	0	0	0	0	1	0	0	1	0	0	1	0	0	0	1	1	1	0	1	0	0	0	0	1	0	1
Helmet		1	0	1	0	0	1	0	0	0	0	0	0	0	0	1	0	0	0	0	0	0	0	0	0	0	1	0	0	0	0	0	0	0	1	0	0
Shield		0	0	1	0	0	0	0	0	0	0	0	0	0	0	0	0	0	0	0	0	0	0	0	0	0	1	1	0	0	0	0	0	0	1	0	0
Whorls		0	1	0	1	0	0	1	0	1	0	0	0	1	0	0	0	0	0	1	1	1	0	0	0	0	0	0	0	0	1	0	1	0	0	0	0
bone distaff		0	1	0	0	0	0	1	0	1	0	0	0	0	0	0	0	0	0	1	0	0	0	0	0	0	0	0	0	0	0	0	0	0	0	0	0
iron shear		0	0	1	0	1	0	0	1	0	0	0	0	0	0	1	0	0	0	0	0	0	0	0	0	1	1	1	0	1	0	0	0	0	1	0	0
Razor		0	0	1	0	1	0	0	1	0	0	0	0	0	1	1	0	0	1	0	0	0	0	0	0	0	0	0	0	0	0	0	0	0	0	0	0
bronze colum		0	0	1	0	1	0	0	0	0	0	0	0	0	0	0	0	0	0	0	0	0	0	0	0	0	0	0	0	0	0	0	0	0	1	0	0
iron spit		0	0	0	0	0	0	0	1	0	0	0	0	0	1	0	0	0	0	0	0	0	0	0	0	0	0	0	0	0	0	0	0	0	1	0	0
iron knife		0	0	0	0	0	0	0	1	0	0	0	0	0	1	0	0	0	0	0	0	0	0	0	0	1	0	0	0	0	0	0	0	0	1	0	0
bronze grater		0	0	0	0	0	0	0	1	0	0	0	0	0	0	0	0	0	0	0	0	0	0	0	0	0	0	0	0	0	0	0	0	0	0	0	0
bell-shaped lid		0	0	0	0	0	0	0	0	1	0	0	0	0	0	0	0	0	0	0	0	0	0	0	0	0	0	0	0	0	0	0	0	0	0	0	0
Whetstone		0	0	0	0	0	0	0	0	0	0	0	0	0	1	0	0	0	0	0	0	0	0	0	0	0	0	0	0	0	0	0	0	0	0	0	0
iron hoe		0	0	0	0	0	0	0	0	0	0	0	0	0	0	0	0	0	0	1	0	0	0	0	0	0	0	0	0	0	0	0	0	0	0	0	0
Strigil		1	0	1	0	1	0	0	0	0	0	0	0	0	0	0	0	0	1	0	0	0	0	0	0	0	1	1	0	0	0	0	0	0	1	0	1
bronze vase		1	0	1	0	1	0	0	0	1	0	0	0	0	0	0	0	0	1	0	0	0	0	0	0	0	1	1	0	0	0	0	0	0	1	0	1
black-glazed aryballos		0	1	0	0	0	0	0	0	0	0	0	0	0	0	0	0	0	0	0	0	0	0	0	0	0	0	0	0	0	0	0	0	0	0	0	0
Amphoriskos		0	0	0	0	0	0	1	0	0	0	0	0	0	0	0	0	0	0	0	0	0	0	0	0	0	0	0	0	0	0	0	0	0	0	0	0
Mirror		0	1	0	0	0	0	1	0	0	0	0	0	0	0	0	0	0	0	1	0	0	0	0	0	0	0	0	0	0	0	0	0	0	0	0	0
grooming tools		0	0	0	0	0	0	0	0	0	0	1	0	0	0	0	0	0	0	0	0	0	0	0	0	0	0	0	0	0	0	0	0	0	0	0	0
amber pearl		1	1	0	1	0	0	1	0	0	0	0	0	0	0	0	0	0	0	0	0	0	0	0	0	0	0	0	0	0	0	0	0	0	0	0	0
glass paste pearl		0	0	0	0	0	0	1	0	0	0	0	0	0	0	0	0	0	0	1	0	0	0	0	0	0	0	0	0	0	0	0	1	1	0	0	0
bone pearl		0	0	0	0	0	0	0	0	0	0	0	0	1	0	0	0	0	0	0	0	0	0	0	0	0	0	0	0	0	0	0	0	0	0	0	0
Pendant		0	1	1	0	0	0	1	0	0	0	0	0	0	0	0	0	0	0	0	0	0	0	0	0	0	0	0	0	0	0	0	0	0	0	1	0
Fibula		0	1	1	0	1	0	1	1	1	0	0	1	1	0	1	0	0	0	1	0	1	0	0	1	0	1	0	1	1	1	1	1	0	0	0	1
Ring		0	0	0	0	0	0	0	0	1	0	0	0	1	1	0	0	0	0	1	0	0	0	0	1	0	0	0	0	0	0	0	0	0	0	0	0
iron bracelet		0	0	0	0	0	0	0	0	0	0	1	0	0	0	0	0	0	0	0	0	0	0	0	1	0	0	0	0	0	0	0	0	0	0	0	0
Candelabrum		1	0	0	0	1	0	1	1	0	0	0	0	1	1	0	0	0	1	0	0	0	0	1	0	1	1	1	0	1	1	1	0	0	1	0	1
Lophos		1	0	0	0	0	0	0	0	0	0	0	0	0	0	0	0	0	0	0	0	0	0	0	0	0	0	0	0	0	0	0	0	0	0	0	0
aes rude		1	0	1	0	1	0	0	0	0	0	0	0	0	0	0	0	0	0	0	0	0	0	0	0	0	0	0	0	0	0	0	1	0	1	0	0
cereal seeds		0	1	0	0	0	0	0	0	0	0	0	0	0	0	0	0	0	0	0	0	0	0	0	0	0	0	0	0	0	0	0	0	0	0	0	0
sea shells		0	0	0	0	1	0	0	0	0	0	0	0	0	0	0	0	0	0	0	0	0	0	0	0	0	0	0	0	0	0	0	0	0	0	0	0
bronze leaf		0	0	0	0	0	0	0	0	0	0	0	0	0	0	0	0	0	0	0	0	0	0	0	0	0	0	1	0	0	0	0	0	0	0	0	0
iron cylinder		0	0	0	0	0	0	0	0	0	0	0	0	0	0	0	0	0	0	0	0	0	0	0	0	0	0	0	0	0	1	0	0	0	0	0	0
Firedog		0	0	0	0	0	0	0	0	0	0	0	0	0	0	0	0	0	0	0	0	0	0	0	0	0	0	0	0	0	0	0	0	0	1	0	0

[1]Origins based on isotopic results: 0=local, 1= missing data, 2= non local.
