# Supplementary material for: Unravelling biocultural population structure in 4th/3rd century BC Monterenzio Vecchio (Bologna, Italy) through a comparative analysis of strontium isotopes, non-metric dental evidence, and funerary practices
Source: PLoS One. 2018 Mar 28;13(3):e0193796. doi: 10.1371/journal.pone.0193796 (PMC5874009; doi:10.1371/journal.pone.0193796)
Supplement: S2 Table — (PDF) [file pone.0193796.s008.pdf]

S2 Table. Correspondence Analysis on age classes.

Principal inertias (eigenvalues)			
**dim**	**value**	**%**	**cum%**
1	0,319158	35,1	35,1
2	0,257762	28,3	63,4
3	0,160431	17,6	81,1
4	0,10928	12	93,1
5	0,063094	6,9	100
--------	-----		
Total:	0,909726	100	

**Rows:**	**name**	**mass**	**qlt**	**inr**	**k=1**	**cor**	**ctr**	**k=2**	**cor**	**ctr**
1	I	11	847	238	-1576	129	87	3721	718	604
2	C	101	521	162	-628	271	125	604	250	143
3	YA	267	726	170	637	699	339	125	27	16
4	A	73	148	83	379	139	33	96	9	3
5	MA	287	116	132	211	106	40	-65	10	5
6	OA	261	920	214	-677	615	376	-476	304	230

**Columns:**	**name**	**mass**	**qlt**	**inr**	**k=1**	**cor**	**ctr**	**k=2**	**cor**	**ctr**
1	skyphos	31	881	35	-870	743	73	-376	138	17
2	trefoil oinochoe	3	63	8	374	56	1	-128	7	0
3	kylix	37	930	17	623	929	44	6	0	0
4	mortar	22	815	11	591	815	25	-3	0	0
5	pitcher	3	820	9	-1199	508	13	-939	311	10
6	kelébe	3	484	8	1127	462	11	245	22	1
7	kantharos	8	186	9	-150	23	1	-398	162	5
8	ceramic kyathos	17	610	5	412	585	9	-86	25	0
9	krater	8	828	10	-675	418	12	-668	410	15
10	globular vase (figuline)	3	298	27	-1111	139	11	1189	159	15
11	jar (bucchero)	3	484	8	1127	462	11	245	22	1
12	stamons jar	8	371	2	101	44	0	-274	327	2
13	jar	45	437	6	212	399	6	-65	37	1
14	black-glazed bowl	25	118	3	110	106	1	-37	12	0
15	bowl	67	186	13	-181	181	7	30	5	0
16	bowl (bucchero)	53	894	13	-129	72	3	433	822	39
17	stemmed cup	3	63	8	374	56	1	-128	7	0
18	painted cup	8	186	9	-150	23	1	-398	162	5
19	cup	11	868	17	-806	473	23	-736	395	24
20	cup (bucchero)	8	67	15	-121	9	0	311	59	3
21	black-glazed plate	6	820	17	-1199	508	25	-939	311	19
22	stemmed plate	28	153	15	37	3	0	-270	150	8
23	plate (bucchero)	39	54	10	-66	20	1	87	34	1
24	plate	22	48	3	77	43	0	-26	5	0
25	glass	62	106	14	-51	13	1	138	93	5
26	miniaturized vase	14	912	231	-1486	148	97	3382	764	623
27	bronze kyathos	8	371	2	101	44	0	-274	327	2
28	situla	3	820	9	-1199	508	13	-939	311	10
29	iron sword with scabbard	28	781	32	885	756	69	160	25	3
30	bronze belt rings	11	692	12	825	673	24	138	19	1
31	iron chain belt	3	484	8	1127	462	11	245	22	1
32	iron javelin	22	640	34	919	615	59	185	25	3
33	iron spear	22	692	25	825	673	48	138	19	2
34	helmet	11	700	10	751	696	20	59	4	0
35	shield	8	743	10	876	729	20	121	14	0
36	whorls	28	899	23	-689	629	42	-451	270	22
37	bone distaff	11	868	17	-806	473	23	-736	395	24
38	iron shear	20	805	7	515	800	16	-38	4	0
39	razor	14	482	5	361	418	6	-141	64	1
40	bronze colum	8	371	2	101	44	0	-274	327	2
41	iron spit	8	63	23	374	56	4	-128	7	1
42	iron knife	11	265	15	562	264	11	-35	1	0
43	bronze grater	3	63	8	374	56	1	-128	7	0
44	bell-shaped lid	3	820	9	-1199	508	13	-939	311	10
45	whetstone	3	63	8	374	56	1	-128	7	0
46	iron hoe	3	820	9	-1199	508	13	-939	311	10
47	strigil	20	574	13	580	573	21	-30	2	0
48	bronze vase	22	376	10	357	324	9	-144	53	2
49	black-glazed aryballos	3	63	8	374	56	1	-128	7	0
50	amphoriskos	3	820	9	-1199	508	13	-939	311	10

51	mirror	8	828	10	-675	418	12	-668	410	15
52	grooming tools	3	484	8	1127	462	11	245	22	1
53	amber pearl	11	548	10	-413	205	6	-533	343	12
54	glass paste pearl	11	556	30	-1155	549	47	125	6	1
55	bone pearl	3	820	9	-1199	508	13	-939	311	10
56	pendant	11	159	4	-202	132	1	92	27	0
57	fibulae	48	766	23	-462	476	32	360	290	24
58	ring	14	958	14	-867	823	33	-351	135	7
59	iron bracelet	6	213	15	8	0	0	717	213	11
60	candelabrum	39	913	1	55	128	0	-136	785	3
61	lophos	3	63	8	374	56	1	-128	7	0
62	aes rude	14	38	4	-87	30	0	48	9	0
63	cereal seeds	3	63	8	374	56	1	-128	7	0
64	sea shells	3	820	9	-1199	508	13	-939	311	10
65	iron cylinder	3	820	9	-1199	508	13	-939	311	10
66	firedog	3	63	8	374	56	1	-128	7	0
