# Supplementary material for: Unravelling biocultural population structure in 4th/3rd century BC Monterenzio Vecchio (Bologna, Italy) through a comparative analysis of strontium isotopes, non-metric dental evidence, and funerary practices
Source: PLoS One. 2018 Mar 28;13(3):e0193796. doi: 10.1371/journal.pone.0193796 (PMC5874009; doi:10.1371/journal.pone.0193796)
Supplement: S3 Table — (PDF) [file pone.0193796.s009.pdf]

# S3 Table. Correspondence Analysis on provenance.

| | Principal inertias (eigenvalues) | | |
|---|---|---|---|
| **dim** | **value** | **%** | **cum%** |
| 1 | 0,132878 | 56,1 | 56,1 |
| 2 | 0,10413 | 43,9 | 100 |
| -------- | ----- | | |
| Total: | 0,237008 | 100 | |

| **Rows:** | **name** | **mass** | **qlt** | **inr** | **k=1** | **cor** | **ctr** | **k=2** | **cor** | **ctr** |
|---|---|---|---|---|---|---|---|---|---|---|
| 1 | 0 | 357 | 1000 | 360 | -486 | 990 | 635 | -49 | 10 | 8 |
| 2 | 1 | 297 | 1000 | 338 | 326 | 393 | 237 | -405 | 607 | 467 |
| 3 | 2 | 347 | 1000 | 303 | 222 | 238 | 128 | 397 | 762 | 525 |

| **Columns:** | **name** | **mass** | **qlt** | **inr** | **k=1** | **cor** | **ctr** | **k=2** | **cor** | **ctr** |
|---|---|---|---|---|---|---|---|---|---|---|
| 1 | skyphos | 27 | 1000 | 4 | 182 | 930 | 7 | -50 | 70 | 1 |
| 2 | trefoil oinochoe | 2 | 1000 | 19 | -1334 | 987 | 33 | -153 | 13 | 1 |
| 3 | kylix | 37 | 1000 | 12 | 18 | 4 | 0 | 273 | 996 | 27 |
| 4 | mortar | 25 | 1000 | 11 | 82 | 63 | 1 | 319 | 937 | 24 |
| 5 | pitcher | 5 | 1000 | 12 | 751 | 1000 | 21 | -12 | 0 | 0 |
| 6 | kelébe | 2 | 1000 | 19 | -1334 | 987 | 33 | -153 | 13 | 1 |
| 7 | kantharos | 7 | 1000 | 57 | -1334 | 987 | 100 | -153 | 13 | 2 |
| 8 | ceramic kyathos | 17 | 1000 | 2 | 135 | 535 | 2 | 126 | 465 | 3 |
| 9 | krater | 7 | 1000 | 26 | 151 | 28 | 1 | -887 | 972 | 57 |
| 10 | globular vase (figuline) | 2 | 1000 | 19 | -1334 | 987 | 33 | -153 | 13 | 1 |
| 11 | jar (bucchero) | 2 | 1000 | 20 | 608 | 196 | 7 | 1231 | 804 | 36 |
| 12 | stamons jar | 7 | 1000 | 18 | -687 | 832 | 27 | 308 | 168 | 7 |
| 13 | jar | 50 | 1000 | 3 | 28 | 49 | 0 | -123 | 951 | 7 |
| 14 | black-glazed bowl | 25 | 1000 | 1 | -83 | 600 | 1 | -68 | 400 | 1 |
| 15 | bowl | 65 | 1000 | 3 | 98 | 854 | 5 | 41 | 146 | 1 |
| 16 | bowl (bucchero) | 52 | 1000 | 19 | 227 | 585 | 20 | 191 | 415 | 18 |
| 17 | stemmed cup | 2 | 1000 | 25 | 893 | 337 | 15 | -1254 | 663 | 38 |
| 18 | painted cup | 10 | 1000 | 8 | 265 | 355 | 5 | -357 | 645 | 12 |
| 19 | cup | 10 | 1000 | 18 | -363 | 312 | 10 | 539 | 688 | 28 |
| 20 | cup (bucchero) | 7 | 1000 | 57 | -1334 | 987 | 100 | -153 | 13 | 2 |
| 21 | black-glazed plate | 5 | 1000 | 9 | -363 | 312 | 5 | 539 | 688 | 14 |
| 22 | stemmed plate | 27 | 1000 | 6 | 156 | 441 | 5 | 176 | 559 | 8 |
| 23 | plate (bucchero) | 37 | 1000 | 10 | -241 | 882 | 16 | 88 | 118 | 3 |
| 24 | plate | 20 | 1000 | 1 | -49 | 226 | 0 | 91 | 774 | 2 |
| 25 | glass | 60 | 1000 | 5 | -118 | 738 | 6 | -70 | 262 | 3 |
| 26 | miniaturized vase | 15 | 1000 | 90 | 522 | 192 | 31 | -1070 | 808 | 165 |
| 27 | bronze kyathos | 7 | 1000 | 0 | 56 | 473 | 0 | -59 | 527 | 0 |
| 28 | situla | 2 | 1000 | 20 | 608 | 196 | 7 | 1231 | 804 | 36 |
| 29 | iron sword with scabbard | 32 | 1000 | 2 | 98 | 854 | 2 | 41 | 146 | 1 |
| 30 | bronze belt rings | 15 | 1000 | 11 | 379 | 830 | 16 | 172 | 170 | 4 |
| 31 | iron chain belt | 2 | 1000 | 19 | -1334 | 987 | 33 | -153 | 13 | 1 |
| 32 | iron javelin | 30 | 1000 | 6 | 218 | 937 | 11 | 57 | 63 | 1 |
| 33 | iron spear | 25 | 1000 | 2 | 111 | 714 | 2 | 70 | 286 | 1 |
| 34 | helmet | 15 | 1000 | 15 | 103 | 45 | 1 | -473 | 955 | 32 |
| 35 | shield | 10 | 1000 | 8 | 265 | 355 | 5 | -357 | 645 | 12 |
| 36 | whorls | 25 | 1000 | 12 | 334 | 986 | 21 | -40 | 14 | 0 |
| 37 | bone distaff | 10 | 1000 | 4 | -292 | 926 | 6 | -82 | 74 | 1 |
| 38 | iron shear | 22 | 1000 | 1 | 56 | 473 | 1 | -59 | 527 | 1 |
| 39 | razor | 15 | 1000 | 8 | 8 | 1 | 0 | 355 | 999 | 18 |
| 40 | bronze colum | 7 | 1000 | 0 | 56 | 473 | 0 | -59 | 527 | 0 |
| 41 | iron spit | 7 | 1000 | 0 | 56 | 473 | 0 | -59 | 527 | 0 |
| 42 | iron knife | 10 | 1000 | 5 | 194 | 351 | 3 | 264 | 649 | 7 |
| 43 | bronze grater | 2 | 1000 | 19 | -1334 | 987 | 33 | -153 | 13 | 1 |
| 44 | bell-shaped lid | 2 | 1000 | 25 | 893 | 337 | 15 | -1254 | 663 | 38 |
| 45 | whetstone | 2 | 1000 | 20 | 608 | 196 | 7 | 1231 | 804 | 36 |
| 46 | iron hoe | 2 | 1000 | 20 | 608 | 196 | 7 | 1231 | 804 | 36 |
| 47 | strigil | 20 | 1000 | 1 | -49 | 226 | 0 | 91 | 774 | 2 |
| 48 | bronze vase | 22 | 1000 | 1 | 56 | 473 | 1 | -59 | 527 | 1 |
| 49 | black-glazed aryballos | 2 | 1000 | 19 | -1334 | 987 | 33 | -153 | 13 | 1 |
| 50 | amphoriskos | 2 | 1000 | 19 | -1334 | 987 | 33 | -153 | 13 | 1 |
| 51 | mirror | 7 | 1000 | 18 | -687 | 832 | 27 | 308 | 168 | 7 |
| 52 | grooming tools | 2 | 1000 | 20 | 608 | 196 | 7 | 1231 | 804 | 36 |
| 53 | amber pearl | 10 | 1000 | 33 | -778 | 767 | 45 | -428 | 233 | 18 |

| | | | | | | | | | | |
|---|---|---|---|---|---|---|---|---|---|---|
| 54 | glass paste pearl | 10 | 1000 | 4 | -292 | 926 | 6 | -82 | 74 | 1 |
| 55 | bone pearl | 2 | 1000 | 25 | 893 | 337 | 15 | -1254 | 663 | 38 |
| 56 | pendant | 10 | 1000 | 33 | -778 | 767 | 45 | -428 | 233 | 18 |
| 57 | fibulae | 47 | 1000 | 8 | -3 | 0 | 0 | -194 | 1000 | 17 |
| 58 | ring | 12 | 1000 | 6 | 334 | 986 | 10 | -40 | 14 | 0 |
| 59 | iron bracelet | 5 | 1000 | 9 | -363 | 312 | 5 | 539 | 688 | 14 |
| 60 | candelabrum | 40 | 1000 | 2 | -49 | 226 | 1 | 91 | 774 | 3 |
| 61 | lophos | 2 | 1000 | 19 | -1334 | 987 | 33 | -153 | 13 | 1 |
| 62 | aes rude | 12 | 1000 | 5 | -55 | 29 | 0 | -317 | 971 | 12 |
| 63 | cereal seeds | 2 | 1000 | 19 | -1334 | 987 | 33 | -153 | 13 | 1 |
| 64 | sea shells | 2 | 1000 | 20 | 608 | 196 | 7 | 1231 | 804 | 36 |
| 65 | bronze leaf | 2 | 1000 | 25 | 893 | 337 | 15 | -1254 | 663 | 38 |
| 66 | iron cylinder | 2 | 1000 | 20 | 608 | 196 | 7 | 1231 | 804 | 36 |
| 67 | firedog | 2 | 1000 | 25 | 893 | 337 | 15 | -1254 | 663 | 38 |
