# Supplementary material for: Unravelling biocultural population structure in 4th/3rd century BC Monterenzio Vecchio (Bologna, Italy) through a comparative analysis of strontium isotopes, non-metric dental evidence, and funerary practices
Source: PLoS One. 2018 Mar 28;13(3):e0193796. doi: 10.1371/journal.pone.0193796 (PMC5874009; doi:10.1371/journal.pone.0193796)
Supplement: S5 Table — (PDF) [file pone.0193796.s011.pdf]

S5 Table. Subset of 17 adult individuals and 41 uncorrelated variables used in Random Forest analysis.

Variables	MV	1	2	3	5	7	8	12	15	19	20	21	22	26	27	30	31	39
trefoil oinochoe		0	1	0	0	0	0	0	0	0	0	0	0	0	0	0	0	0
pitcher		0	0	0	1	0	0	0	0	0	0	0	0	0	0	0	0	0
kelébe		0	0	1	0	0	0	0	0	0	0	0	0	0	0	0	0	0
kantharos		0	0	1	0	0	0	0	0	0	0	0	0	0	0	0	0	0
ceramic kyathos		0	0	0	0	0	1	0	0	1	0	1	0	1	0	1	0	0
krater		0	0	0	0	1	0	0	0	0	0	0	0	0	0	0	0	0
jar (bucchero)		0	0	0	0	0	0	0	0	0	0	0	0	0	1	0	0	0
stamons jar		0	0	1	0	0	1	0	0	0	0	0	0	0	0	0	1	0
jar		1	1	1	0	0	1	1	1	1	1	1	1	0	0	1	0	1
black-glazed bowl		1	1	1	1	1	0	0	0	0	0	0	0	1	1	0	0	0
bowl		0	0	1	1	1	1	1	1	1	1	1	1	1	1	1	1	1
painted cup		1	0	0	1	0	0	0	0	0	0	0	0	0	0	0	0	0
cup		1	0	0	1	1	0	0	0	0	0	0	0	0	0	0	1	0
cup (bucchero)		1	1	0	0	0	0	0	0	0	0	0	0	0	0	0	0	0
black-glazed plate		0	0	0	1	1	0	0	0	0	0	0	0	0	0	0	0	0
stemmed plate		1	1	0	1	0	0	0	1	0	1	0	1	0	1	1	0	0
plate (bucchero)		1	1	0	0	0	1	1	0	1	0	1	0	1	0	0	1	1
plate		0	1	1	0	0	1	0	0	1	0	0	0	1	0	0	1	0
miniaturized vase		0	1	0	0	0	0	0	0	0	0	0	0	0	0	0	0	0
bronze kyathos		0	0	1	1	0	0	0	0	0	0	0	0	0	0	0	0	0
situla		0	0	0	1	0	0	0	0	0	0	0	0	0	0	0	0	0
iron chain belt		0	0	1	0	0	0	0	0	0	0	0	0	0	0	0	0	0
helmet		1	0	1	0	0	0	0	0	0	0	0	0	0	1	0	0	0
shield		0	0	1	0	0	0	0	0	0	0	0	0	0	1	0	0	0
bronze colum		0	0	1	1	0	0	0	0	0	0	0	0	0	0	0	0	0
bronze grater		0	0	0	0	0	1	0	0	0	0	0	0	0	0	0	0	0
whetstone		0	0	0	0	0	0	0	1	0	0	0	0	0	0	0	0	0
iron hoe		0	0	0	0	0	0	0	0	0	1	0	0	0	0	0	0	0
black-glazed aryballos		0	1	0	0	0	0	0	0	0	0	0	0	0	0	0	0	0
amphoriskos		0	0	0	0	1	0	0	0	0	0	0	0	0	0	0	0	0
grooming tools		0	0	0	0	0	0	1	0	0	0	0	0	0	0	0	0	0
amber pearl		1	1	0	0	1	0	0	0	0	0	0	0	0	0	0	0	0
glass paste pearl		0	0	0	0	1	0	0	0	0	1	0	0	0	0	0	0	0
pendant		0	1	1	0	1	0	0	0	0	0	0	0	0	0	0	0	0
fibulae		0	1	1	1	1	1	0	0	0	1	0	1	0	1	1	1	1
iron bracelet		0	0	0	0	0	0	1	0	0	0	0	0	0	0	0	0	0
lophos		1	0	0	0	0	0	0	0	0	0	0	0	0	0	0	0	0
aes rude		1	0	1	1	0	0	0	0	0	0	0	0	0	0	0	0	0
cereal seeds		0	1	0	0	0	0	0	0	0	0	0	0	0	0	0	0	0
sea shells		0	0	0	1	0	0	0	0	0	0	0	0	0	0	0	0	0
iron cylinder		0	0	0	0	0	0	0	0	0	0	0	0	0	0	0	1	0
