# Supplementary material for: Unravelling biocultural population structure in 4th/3rd century BC Monterenzio Vecchio (Bologna, Italy) through a comparative analysis of strontium isotopes, non-metric dental evidence, and funerary practices
Source: PLoS One. 2018 Mar 28;13(3):e0193796. doi: 10.1371/journal.pone.0193796 (PMC5874009; doi:10.1371/journal.pone.0193796)
Supplement: S7 Table — (PDF) [file pone.0193796.s013.pdf]

S7 Table. Confusion Matrix for origin (OOB error=52.94%).

	0	1	class.error
0	1	6	0.86
1	3	7	0.30
